# An “up” oriented methionine-aromatic structural motif in SUMO is critical for its stability and activity

**DOI:** 10.1016/j.jbc.2021.100970

**Published:** 2021-07-15

**Authors:** Kiran Sankar Chatterjee, Ranabir Das

**Affiliations:** National Centre for Biological Sciences, Tata Institute of Fundamental Research, Bengaluru, India

**Keywords:** methionine-aromatic motifs, sulfur-aromatic interaction, structure-function, SUMOylation, SUMO-interacting motif (SIM), CD, circular dichroism, CSP, chemical shift perturbation, DMS, dimethyl sulfide, MD, molecular dynamics, PML, promyelocytic leukemia, RMSF, root mean square fluctuation, SIM, SUMO interacting motif, SUMO, small ubiquitin-like modifier, UBL, ubiquitin-Like

## Abstract

Protein structural bioinformatic analyses suggest preferential associations between methionine and aromatic amino acid residues in proteins. *Ab initio* energy calculations highlight a conformation-dependent stabilizing interaction between the interacting sulfur-aromatic molecular pair. However, the relevance of buried methionine-aromatic motifs to protein folding and function is relatively unexplored. The Small Ubiquitin-Like Modifier (SUMO) is a β-grasp fold protein and a common posttranslational modifier that affects diverse cellular processes, including transcriptional regulation, chromatin remodeling, metabolic regulation, mitosis, and meiosis. SUMO is a member of the Ubiquitin-Like (UBL) protein family. Herein, we report that a highly conserved and buried methionine-phenylalanine motif is a unique signature of SUMO proteins but absent in other homologous UBL proteins. We also detect that a specific “up” conformation between the methionine-phenylalanine pair of interacting residues in SUMO is critical to its β-grasp fold. The noncovalent interactions of SUMO with its ligands are dependent on the methionine–phenylalanine pair. MD simulations, NMR, and biophysical and biochemical studies suggest that perturbation of the methionine-aromatic motif disrupts native contacts, modulates noncovalent interactions, and attenuates SUMOylation activity. Our results highlight the importance of conserved orientations of Met-aromatic structural motifs inside a protein core for its structure and function.

Nonbonded aromatic interactions play a significant role in stabilizing a folded protein structure (1, 2). The strength of these interactions often depends on the nature of the interacting side chains and their orientation (3, 4). Interactions between sulfur-containing cysteine and methionine side chains and aromatic side chains are consistently observed in proteins and at the interfaces of protein–protein complexes (5, 6, 7, 8). The noncovalent sulfur–aryl interactions are known as S-aromatic motifs in proteins. Methionine-aromatic motifs are examples of S-aromatic motifs that provide additional stabilization at a longer distance range than pure hydrophobic interactions (9). The geometry between the interacting Met-aromatic pair is critical in determining its energetic outcome (10, 11). *Ab initio* calculations with the methionine side chain analog dimethyl sulfide (DMS) and isolated aromatic rings have indicated two major “up” and “down” conformations where the “up” orientation is energetically more favorable over “down” conformations by 2 to 3 kcal/mol (9, 12) (Fig. 1*A*). However, the studies involving Met-aromatic interactions are mostly limited to isolated systems and designed model peptides (13). Whether specific conformations of the Met-aromatic motif within the buried core of a protein provide stability and structural specificity is unclear.

**Figure 1:**
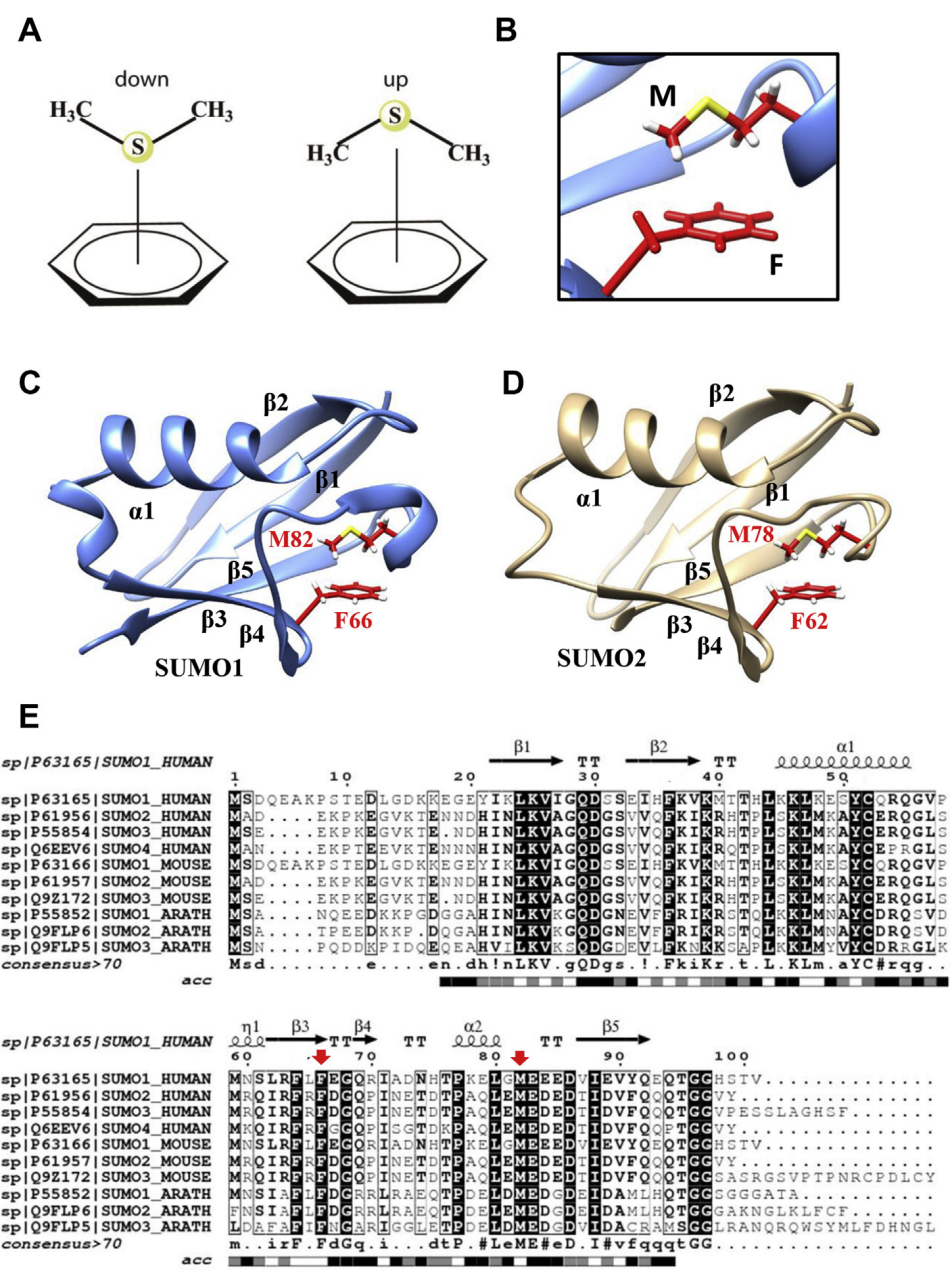
**A methionine–phenylalanine motif is highly conserved in SUMO*.****A*, schematic representation of possible “down” and “up” conformations of dimethyl sulfide interacting with a benzene ring. *B*, zoomed in view at SUMO1 hydrophobic core shows an “up” oriented Met-aromatic motif. *C*, crystal structures of human SUMO1 (4WJQ) (19) (1.35 Å) and *D*, SUMO2 (4NPN) (22) (1.633 Å) are shown, indicating the Met-aromatic motif. Side chains of the interacting residues are shown in *Red*. Sulfur in the methionine side chain is highlighted in *Yellow*. *E*, Multiple Sequence Alignment of SUMO homologs from Human, Mouse, and Arabidopsis. Conserved residues are highlighted in *black*, and *red arrows* mark the conserved methionine and phenylalanine residues. Solvent accessibility is shown at the *bottom* of the alignment on a color scale from *Black* to *White*. *Black* indicates the solvent-exposed residues, and *white* represents buried residues (55). A more comprehensive sequence alignment is provided in Fig. S2.

We investigated a Met-Aromatic motif present in the protein Small Ubiquitin-like Modifier (SUMO), a Ubl (Ubiquitin-like) family member that regulates diverse cellular processes. The Ubl family shares a β-grasp fold where a central helix is packed against a β sheet. Although all Ubls possibly diverged from a common ancestral sequence, they are unique in their function and interactome. The covalent conjugation of SUMO proteins to substrates, known as SUMOylation, involves a multistep enzymatic reaction similar to Ubiquitination (14). SUMOylation involves three distinct steps, namely activation, conjugation, and ligation. During activation, the activating enzyme E1 forms a thioester bond with the C-terminal end of SUMO (15, 16). The activated SUMO is subsequently transferred to the conjugating enzyme E2 (Ubc9), where a catalytic cysteine residue in Ubc9 forms a thioester bond with SUMO (17). The E3 ligases bring the Ubc9∼SUMO conjugate and the substrate close in space to catalyze SUMO ligation to the substrates (18, 19). SUMOylation can also occur without E3 ligases, albeit slower, where SUMO gets directly transferred from the Ubc9∼SUMO complex to a substrate Lysine residue. SUMOylation regulates multiple signaling pathways that are essential for maintaining cellular homeostasis (20).

We report that a conserved “up” oriented Met-Aromatic motif is present at the buried core of SUMO. The motif is present in all SUMO isoforms but absent in other Ubls. A detailed biophysical and biochemical approach was used to study the conserved Met-aromatic motif’s significance for SUMO stability and function. Even slight perturbations of the Met-aromatic motif by replacing methionine with conserved aliphatic residues result in reduced stability, altered structure, and impaired function. Overall, our results strongly suggest conserved “up” oriented Met-aromatic interaction’s relevance for maintaining the protein structure–function relationship.

## Results

### A Met-aromatic motif is conserved across all SUMO isoforms

A methionine residue (M82) interacts with a phenylalanine (F66) aromatic ring in human SUMO1 and SUMO2 (Fig. 1, *A* and *B*). The M82 side chain is oriented in an “up” conformation above the F66 aromatic ring (Fig. 1, *C* and *D*) (21, 22). F66 is present in the beta-strand β3, while M82 is present in the unstructured β3-β5 loop. Interestingly, compared with other Ubiquitin-like protein sequences, we found that the conserved methionine is unique for SUMO and absent for other Ubiquitin-like homologous folds (Fig. S1). In Ubiquitin or NEDD8, which has a structurally similar β grasp fold, methionine is replaced by an isoleucine residue. The F66 is either conserved or substituted with another aromatic amino acid in other Ubls. Multiple Sequence Alignment of SUMO sequences from different organisms reveals conservation of the involved methionine and phenylalanine residues across different SUMO isoforms (Fig. 1*E*). A more comprehensive sequence alignment, including all the curated SUMO sequences from the Uniprot database (Fig. S2), shows that the methionine-aromatic motif is conserved in 44 out of 49 SUMO protein sequences. The high degree of sequence conservation of the Met-Phe pair across all SUMO isoforms underscores the potential implication of the met-aromatic motif toward SUMO protein stability and activity.

### Disruption of the Met-aromatic motif destabilizes SUMO

**Figure 2:**
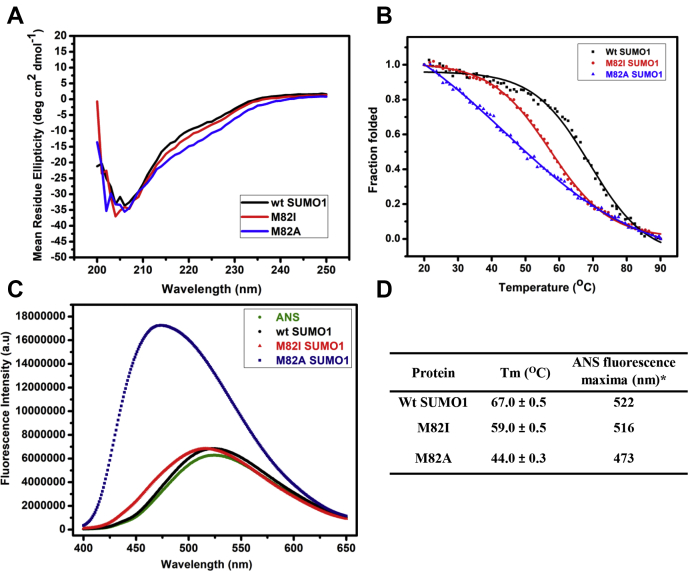
**Biophysical studies of Wt-SUMO1, M82I-SUMO1, and M82A-SUMO1*.****A*, the Far UV Circular Dichroism spectra showed a conserved secondary structure for Wt and SUMO1 mutants. *B*, temperature melts for wt and SUMO1 mutants. Change in mean ellipticity is normalized and plotted against temperature. *C*, the plot of steady-state ANS fluorescence intensity upon binding with Wt and mutant proteins. *D*, Tm and ANS fluorescence maxima for SUMO variants.

Two SUMO1 variants were made where M82 was replaced by isoleucine (M82I) and alanine (M82A) to determine whether the conserved Met-aromatic motif has any role in the stability of SUMO. Isoleucine has similar hydrophobicity to that of methionine and present in structurally similar Ubiquitin fold. Moreover, isoleucine and methionine have the same van der Waals volume of 124 Å^3^ (23). Thus methionine to isoleucine substitution in SUMO1 (M82I) would possibly reveal sulfur-aromatic interaction’s contribution, if any, toward SUMO stability. Wt and variant SUMO1 were expressed in *E. Coli* and purified as described (Experimental procedures). Far UV, circular dichroism scan showed that the secondary structure is preserved for both the methionine variant SUMO1 (Fig. 2*A*).

Thermal melts were carried out with wt-SUMO1, M82A-SUMO1, and M82I-SUMO1. In comparison to wt-SUMO1, the melting temperature (Tm) decreased for both M82I and M82A variants (Fig. 2*B*). The Tm of M82I-SUMO1 was 59 °C, significantly lower than the Tm 67 °C of wt-SUMO1. The Tm was 44 °C for M82A-SUMO1. M82 is located within the β3 to β5 loop region and not within a helix or beta-strand. Since the mutant CD spectra are similar to the wt protein, the reduced stability suggests disruption of the native tertiary structure but not the secondary structure. Steady-state ANS fluorescence measurement can probe the exposed hydrophobic surface in a protein, typically characterized by its blue-shifted emission maxima and increased fluorescence intensity upon binding (24, 25). Deviations from the native structure can be detected using the steady-state ANS fluorescence. ANS fluorescence was unaffected upon binding to wt-SUMO1 (Fig. 2*C*). For M82I-SUMO1, the emission maxima shifted by 6 nm (522 nm–516 nm), suggesting alteration from the wt-SUMO1 packing due to disruption of the Met-aromatic interaction (Fig. 2*D*). For M82A, the ANS intensity of emission maxima increased threefold and blue-shifted by 49 nm (522 nm–473 nm), indicating significant perturbations in the hydrophobic core packing and further deviation from the wild-type fold. Taken together, the decrease in Tm and change in ANS fluorescence for the mutants suggest that the conserved methionine is essential to maintain the native state stability and structure of SUMO.

### Disruption of the Met-aromatic motif induces structural changes in SUMO1

**Figure 3:**
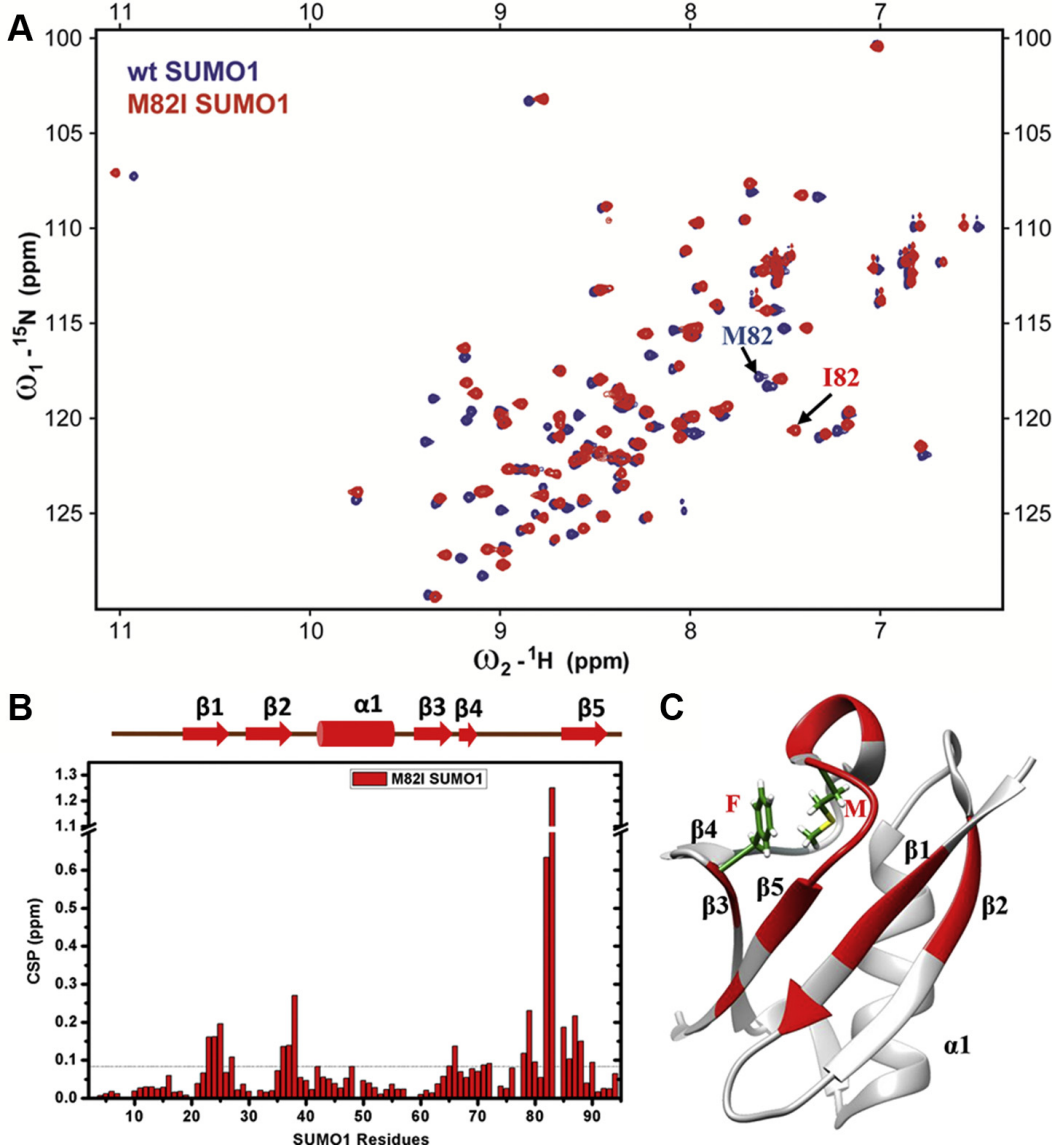
**NMR studies of Wt-SUMO1 and M82I-SUMO1.***A*, overlay of ^15^N-^1^H edited Heteronuclear Single Quantum Coherence (HSQC) spectra of Wt (*Blue*) and M82I-SUMO1 (*Red*). *B*, chemical shift perturbation (CSP) upon mutation plotted against individual residues of M82I-SUMO1. The chemical shift perturbations between the Wt and mutants are calculated as CSP = [(δ^H^_Wt_ − δ^H^_mutant_)^2^+ ((δ^N^_Wt_ − δ^N^_mutant_)/5)^2^]^1/2^ where δ^H^ and δ^N^ are the chemical shift of the amide hydrogen and nitrogen, respectively. The *horizontal line* indicates the mean CSP upon mutation. *C*, residues showing higher than average CSP are mapped in *red* on the SUMO1 structure. The side chains of residues F66 and M82 are shown.

The backbone amide chemical shifts of M82I-SUMO1 were analyzed using solution NMR spectroscopy to assess how disruption of the Met-aromatic motif affects the SUMO1 structure. The wt-SUMO1 and M82I-SUMO1 were expressed in ^13^C, ^15^N-labeled media, and purified (Materials and Methods). ^15^N-^1^H edited Heteronuclear Single Quantum Coherence (HSQC) spectra of M82I-SUMO1 show well-dispersed cross-peaks suggesting a folded protein (Fig. S3). The backbone ^1^H_N_, ^15^N, ^13^C_α_, ^13^C_β_ resonances of the M82I-SUMO1 were assigned by standard triple resonance NMR experiments (Experimental procedures). A comparison of wt-SUMO1 and M82I-SUMO1 ^15^N-^1^H HSQC spectra indicates considerable overlap for a subset of cross-peaks (Fig. 3*A*). However, several peaks between 9 ppm and 10 ppm deviate between the two spectra. Since the backbone amide chemical shifts are sensitive to the chemical environment, the chemical shift perturbation (CSP) plot between M82I-SUMO1 and Wt-SUMO1 resonances would suggest the structural differences between the two proteins. Apart from the site of substitution (M82), significant CSPs were observed across the β1 to β5 region of SUMO1 (Fig. 3, *B* and *C*). The helix in SUMO1 spanning from 44 to 57 residues was unaffected by the substitution (Fig. 3, *B* and *C*). High CSP could also be observed for F66 residue, suggesting a change in its chemical environment upon substitution at M82. There are no direct contacts between the M82 with β1 to β2 strands. Nevertheless, the structural perturbations due to disruption in the M82-Phe66 interaction are transmitted throughout the β-sheet in SUMO1.

### The van der Waals interaction energy of the Met-aromatic motif is significant

**Figure 4:**
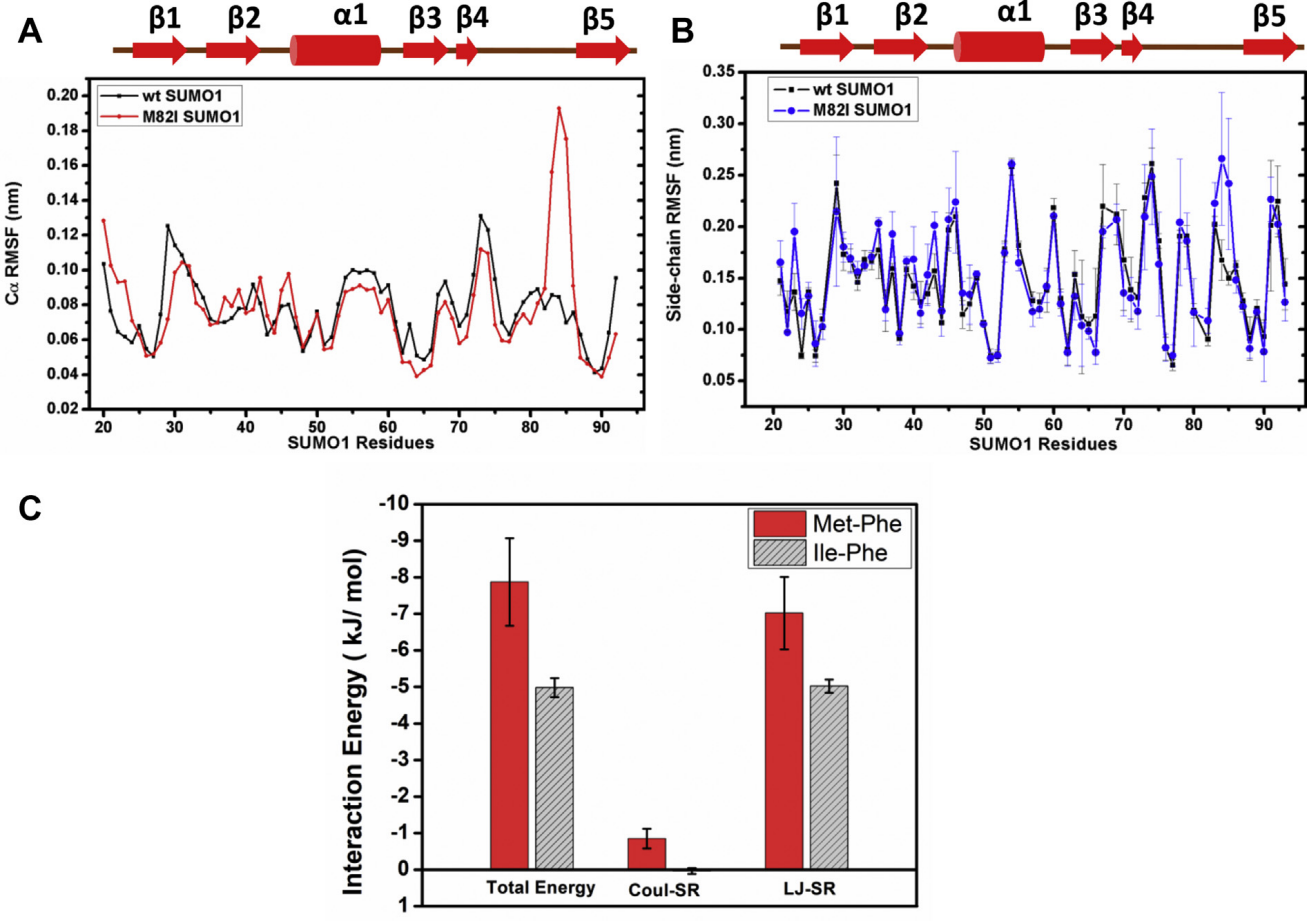
**Comparison of backbone and side chain dynamics of wt-SUMO1 and M82I-SUMO1.***A*, Cα Root Mean Square Fluctuation (RMSF) between wt-SUMO1 (*Black*) and M82I-SUMO1 (*Red*) plotted against SUMO1 residues. *B*, the plot of side chain RMSF between wt-SUMO1 (*Black*) and M82I-SUMO1 (*Blue*). *C*, interaction energy plotted for Met-Phe (*Red*) and Ile-Phe (*Gray*) pair.

All-atom molecular dynamics simulations were carried out with wt and M82I SUMO1 to understand changes in the motional properties induced due to disruption of the Met-aromatic interaction. The Cα Root Mean Square Fluctuation (RMSF) of wt-SUMO1 and M82I-SUMO1 (Fig. 4*A*) reveals a significantly higher fluctuation at the site of substitution (residues: 82–87) (Fig. S4). A slightly higher fluctuation could be observed for residues spanning the β1 region, which agrees with the higher CSPs observed in the NMR spectra. Snapshots at different time points reflect the disruption of native contacts and considerable conformational heterogeneity in the β3 to β5 region. The side chain RMSF plot also shows high fluctuations in these regions (Fig. 4*B*). Notably, the dynamics of the rest of the protein are similar to wt-SUMO1. The conserved Met-aromatic motif in SUMO1 maintains native contacts in the β3 to β5 loop, which is disrupted by aliphatic substitutions.

A total energy function was used on individual all-atom MD simulation trajectories to identify the pairwise interaction energy between M82-F66 side chains (Fig. 4*C* and Fig. S5). On average, methionine stabilizes by ∼−3.0 kJ/mol (∼−0.72 kcal/mol) over isoleucine. The M82–F66 side chain interaction energy is −7.87 kJ/mol (average of three independent runs) (Fig. S6), whereas that for the I82–F66 pair is −4.98 kJ/mol. A small electrostatic component (Coulombic Short Range) contributes to the overall interaction energy for sulfur-containing methionine side chain (∼−0.85 kJ/mol) that is absent in isoleucine. However, the significant contribution is the higher van der Waals interaction energy (∼−2.0 kJ/mol, Lennard-Jones Short Range) of the “up” oriented Met side chain Phenyl aromatic moiety, attributed to the “softer” sulfur lone pair.

### MD simulations suggest structural changes across the β-sheet

**Figure 5:**
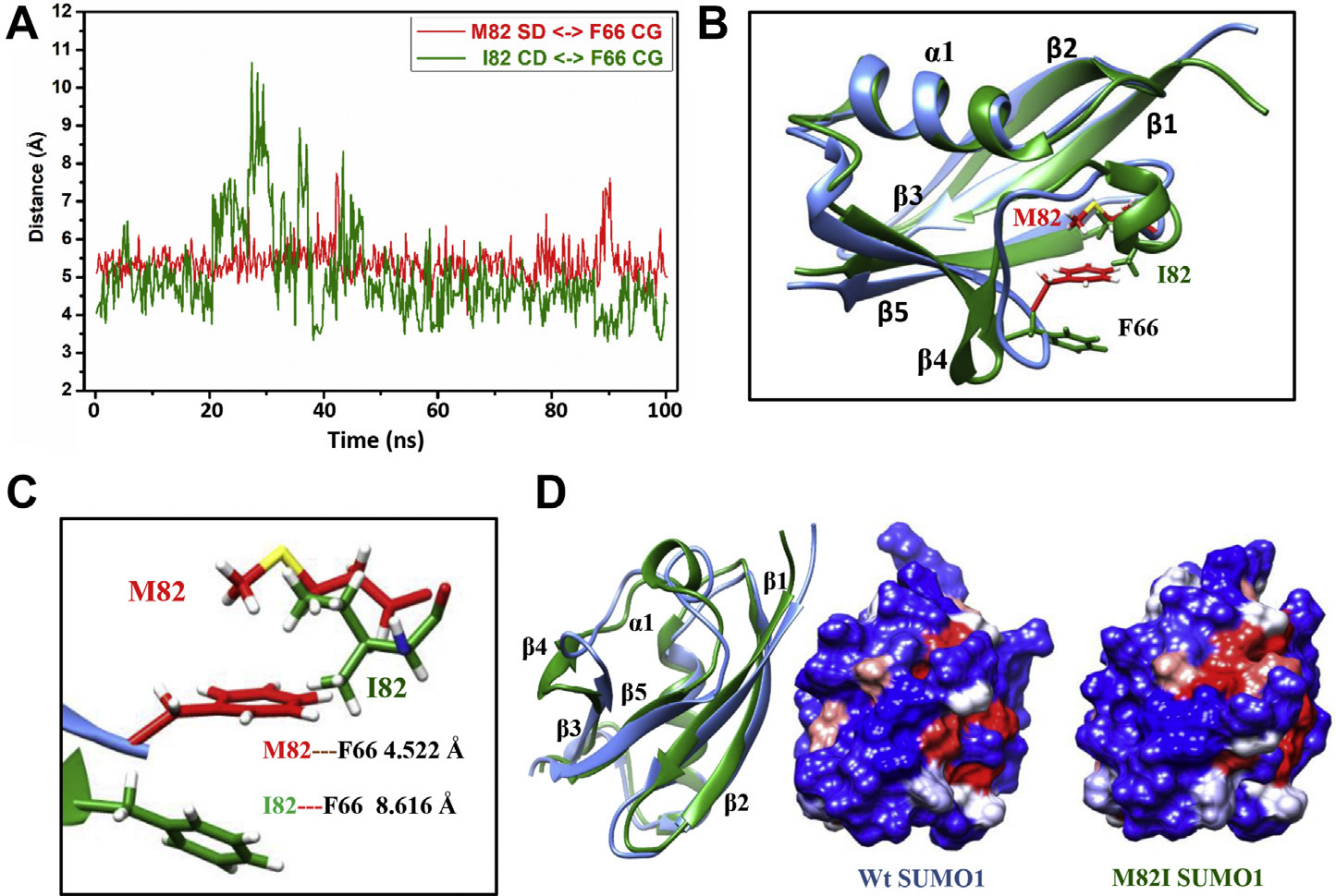
**Perturbation of methionine–phenylalanine motif brings subtle changes in the SUMO1 structure.***A*, distance plot between M82 SD/I82 CD with F66 CG atom. *B*, superposition of wt-SUMO1 (*blue*) and M82I-SUMO1 (*Green*) structures obtained from individual all-atom MD simulation. 144th frame PDBs were used for both wt-SUMO1 and M82I-SUMO1. *C*, zoomed-in view of the M82/I82 -F66 orientations in the same time frame. *D*, hydrophobic surface representation of wt-SUMO1 and M82I-SUMO1 in the same orientation as the overlaid structure. *Red* denotes hydrophobic, and *blue* denotes polar surface.

The simulated trajectories were analyzed to identify possible structural changes in SUMO1 upon M82I substitution. Figure 5*A* represents the distance plot between M82-S/I82-CD with the F66 CG atom. Unlike the M82-F66 pair, where interresidue distance remains constant around 5 Å, the I82–F66 pair shows significant fluctuations. Figure 5*B* represents the overlap of the M82I-SUMO1 structure obtained from all-atom MD simulations with the corresponding wt-SUMO1 (144th frame). Upon structural alignment, a significant deviation was noticed in the β-sheet of M82I-SUMO1. Such a deviation in the β-sheet corroborates the experimentally observed high CSPs in β1, β2, β5, and β3 to β5 loop regions. The β3 to β5 loop in M82I-SUMO1 adopts a more open conformation compared with wt-fold. Comparing contacts between wt-SUMO1 and M82I-SUMO1 structures reveals loss of native contacts due to M82I substitution (Fig. S7). The β2 to β5 contacts, α1 to β3 contacts, and contacts in β3 to β5 regions are altered upon M82I substitution. The distance between the interacting Ile-aromatic pair is much higher (∼8.6 Å) over that of Met-aromatic pair (∼4.5 Å) (Fig. 5*C*), suggesting a drop in favorable sulfur-aromatic interaction. The loss of β3 to β5 loop native contacts in M82I-SUMO1 alters the β-strand’s conformation, coinciding with high CSPs in that region (Fig. 3*B*). The altered conformation of the β sheet in M82I-SUMO1 could alter the interaction interfaces and affect SUMO-mediated noncovalent interactions. Indeed, the electrostatic surface map on individual SUMO structures (Fig. 5*D*) shows the change in M82I-SUMO1 surface compared with wt-SUMO1. Overall, perturbation of the M82–F66 interaction creates multiple structural changes in the protein. The most significant change is in the β-sheet conformation.

### The Met-aromatic motif is critical for SUMO:SIM binding

**Figure 6:**
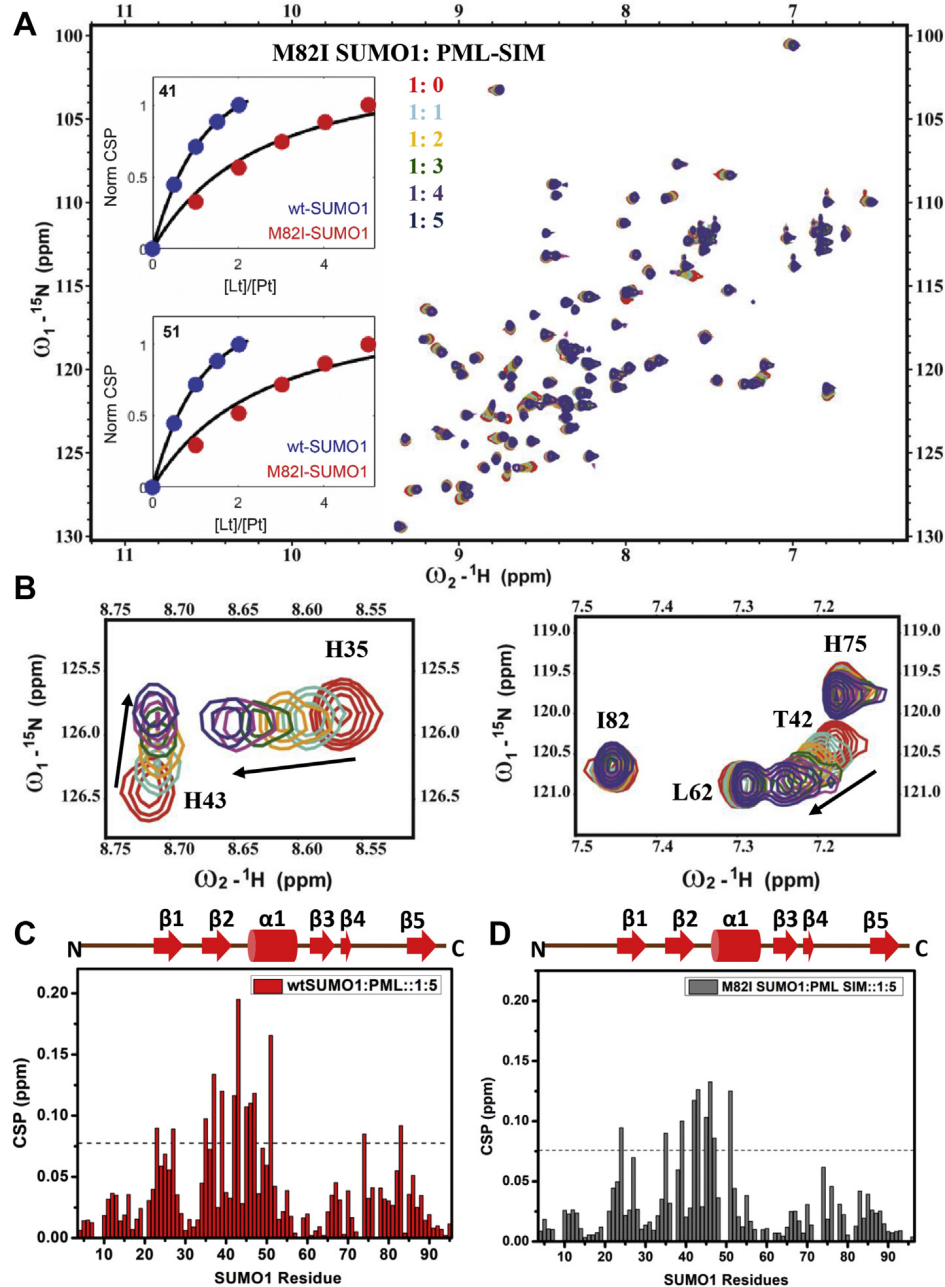
**Interaction of**^**15**^**N M82I-SUMO1 with PML-SIM.***A*, overlay of ^15^N-^1^H edited HSQC spectrum of M82I SUMO1 (*Red*) at different concentrations of PML-SIM. Apo spectrum is shown in *Red*, and the highest Protein/Ligand ratio of 1:5 is shown in *Blue*. A typical fit for Kd determination is shown for two residues in the *inset*. The residue number is provided on the *top left corner* of the *inset*. The Kd values were obtained as averages over individual fits for interface residues in WT-SUMO1 and M82I-SUMO1. *B*, zoomed-in view of some of the amide cross-peaks shows a gradual shift with increasing SIM concentration. The residues present at the SIM binding interface shift with increasing concentration of PML-SIM. Chemical shift perturbation (CSP) plotted against SUMO1 residues upon PML SIM binding. *C*, wt-SUMO1. *D*, M82I-SUMO1. The chemical shift perturbations between the free and PML-SIM bound form are calculated as CSP = [(δ^H^_free_ − δ^H^_bound_)^2^+ ((δ^N^_free_ − δ^N^_bound_)/5)^2^]^1/2^ where δ^H^ and δ^N^ are the chemical shift of the amide hydrogen and nitrogen, respectively. The *dashed line* indicates the mean + SD of CSP values for wt-SUMO1. The secondary structure alignment of SUMO1 against its sequence is provided on *top*.

Noncovalent interaction between SUMO and SUMO Interacting Motif (SIM) is essential for SUMO signaling. The *in-trans* SUMO:SIM interaction between the SUMO conjugated Promyelocytic Leukemia (PML) protein and the PML-SIM creates the phase-separated PML nuclear bodies (26). The SIM binds to the β2-α1 hydrophobic groove within SUMO (Fig. S8*A*). Modeling the SUMO1:SIM complex suggests that due to the shift in β-sheet conformation by M82I substitution, there is a significant gap in the M82I-SUMO1:PML-SIM complex compared with the wt-SUMO1:PML-SIM complex (Fig. S8*B*). Consequently, compared with 100 contacts in the wt-SUMO1:PML-SIM complex, the M82I-SUMO1:PML-SIM complex has 73 contacts, suggesting that the affinity for PML-SIM for SUMO1 may be reduced due to M82I substitution. NMR titrations were carried out for the SUMO1:PML-SIM complex to see whether disruption of the Met-aromatic motif in SUMO impacts SIM binding. A peptide of PML-SIM sequence was titrated against ^15^N-labeled wt-SUMO1, and ^15^N-^1^H edited HSQCs were recorded at each protein:ligand ratio (Fig. 6, *A* and *B*). Figure 6*C* shows the CSP plot against residues for wt-SUMO1 upon PML-SIM binding. Significant perturbations at the β2-α1 region indicate the canonical SUMO:SIM interaction. A similar titration was performed for the M82I-SUMO1:PML-SIM complex. At equivalent SUMO:SIM ratio (1:5), the CSP patterns of wt-SUMO1 and M82I-SUMO1 are similar, suggesting that the binding interface is similar (Fig. 6, *C* and *D*). However, M82I-SUMO1 shows lower CSPs in the β2-α1 region (Fig. 6*D*), suggesting that the SIM binding interface is affected in M82I-SUMO1. Fitting the NMR titration curves yields the dissociation constants, which indicate a ∼ sixfold reduction of binding affinity for M82I-SUMO1 (Kd∼ 568 ± 40 μM) (Fig. S9) in comparison to wt-SUMO1 (Kd∼ 85 ± 15 μM).

### The Met-aromatic motif supports Ubc9:SUMO noncovalent interaction

**Figure 7:**
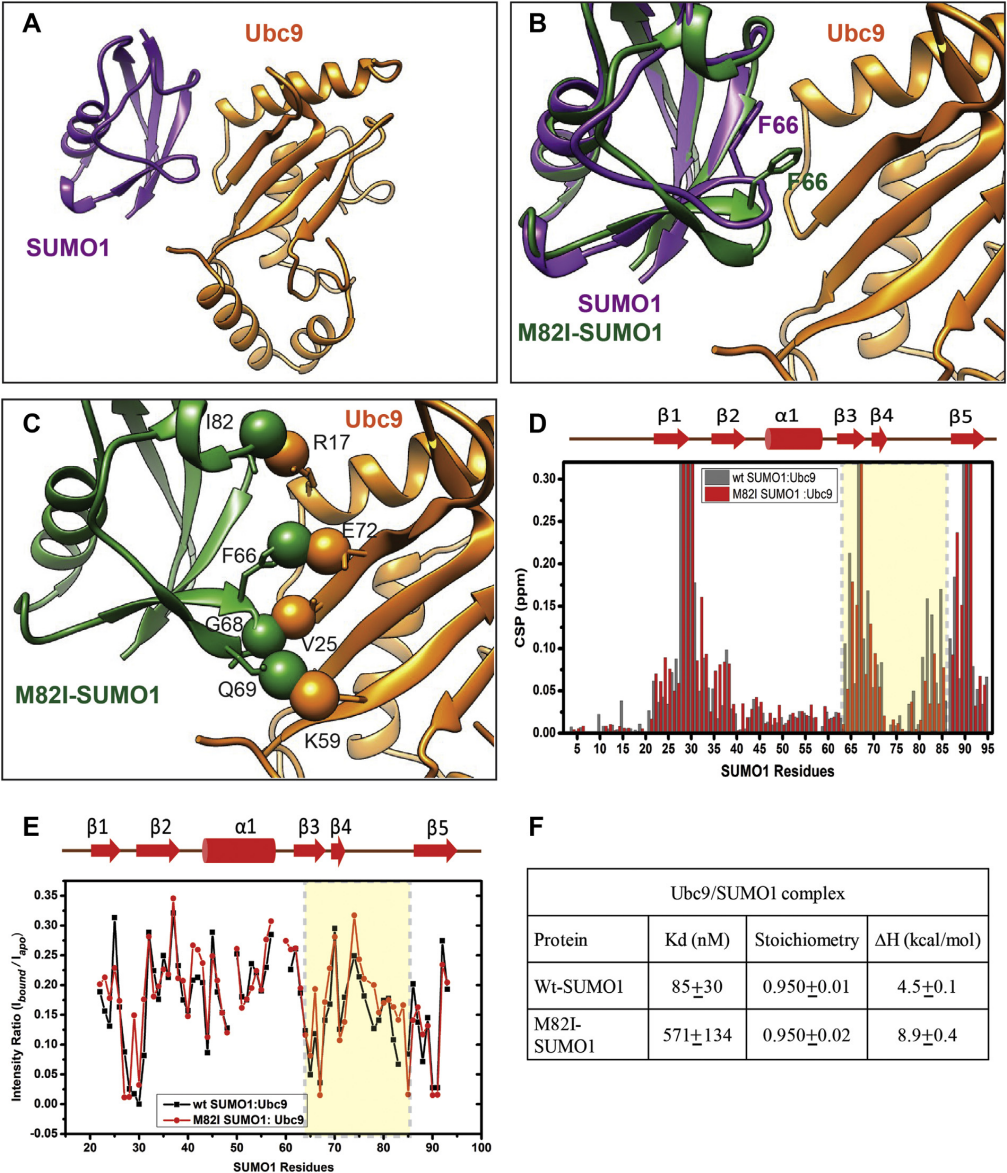
**The methionine–phenylalanine motif is important for Ubc9–SUMO1 interaction.***A*, the Ubc9:SUMO1 complex structure (PDB id: 2PE6). *B*, superposition of M82I-SUMO1 on wt-SUMO1 Ubc9:SUMO1 complex. The SUMO1 structures are the same as in Figure 5*B*. *C*, the clashes observed between M82I-SUMO1 atoms and that of Ubc9 atoms are highlighted. The clashing atoms are shown as *spheres*. The relevant residues are mentioned in *black*. *D*, chemical shift perturbation (CSP) upon Ubc9 binding plotted against individual residues of SUMOs. Broadened out residues with undefined CSPs are shown as *vertical bars*. The β3 to β5 region is highlighted in a *light yellow rectangle*. The secondary structure alignment of SUMO1 against its sequence is provided on *top*. *E*, comparison of the I_bound_/I_apo_ ratio, the Wt, and M82I-SUMO1. A higher I_bound_/I_apo_ ratio suggests lesser binding. The β3 to β5 region is highlighted in a *light yellow rectangle*. *F*, a table showing the Kd, Stoichiometry, and enthalpy ΔH of ITC binding studies of wt-SUMO1:Ubc9 and M82I-SUMO1:Ubc9 complex.

A strong noncovalent interaction between SUMO and Ubc9 (SUMO E2) is necessary for efficient SUMOylation (27). The β3 to β5 loop region, β5, and β1 to β2 turn of SUMO form the interface in the noncovalent SUMO1:Ubc9 complex (2UYZ) (Fig. 7*A*) (27). A disruption of the Met-aromatic motif causes significant deviations in the β5 and β3 to β5 loop, which is part of the Ubc9-interacting interface (Figs. 3*B* and 4). A superposition of M82I-SUMO1 on the Ubc9:SUMO1 complex indicates severe clashes between Ubc9 and SUMO1, which is expected to reduce the binding affinity of the complex (Fig. 7, *B* and *C*). We carried out titration experiments by NMR to determine if the Met-aromatic motif influences the Ubc9 interaction. Unlabeled Ubc9 was purified and titrated against ^15^N labeled SUMOs. For both wt- and M82I-SUMO1, a subset of cross-peaks broadened during titration (Fig. S10). The CSP plot shows reduced CSPs in the β3 and β5 loop of M82I-SUMO1compared with wt-SUMO1, suggesting that the Met-aromatic motif impacts the Ubc9 interaction (Fig. 7*D*). To further validate this, the ratio of peak intensities for bound *versus* apo form was plotted for wt- and M82I-SUMO1 (Fig. 7*E*). At 1:1 ratio of SUMO1:Ubc9 concentration, the ratio of I_bound_/I_apo_ was higher in the β3 to β5 loop (75–79, 82–84) for the mutant SUMO1, confirming that disruption of the stable Met-aromatic interaction impairs Ubc9 interaction. The reverse titration was performed, where unlabeled SUMO was added to ^15^N labeled Ubc9 (Fig. S11). Unlike wt-SUMO1, interaction with methionine variant SUMOs (M82I and M82A) showed a lesser broadening of Ubc9 cross-peaks (Fig. S12). Quantification of bound *versus* apo peak intensity ratio (I_bound_/I_apo_) of Ubc9 shows progressively lesser binding from wt-to M82I-, followed by M82A-SUMO1 (Fig. S13). To quantify the strength of binding, the dissociation constant was measured by ITC experiments. The Kd of wt-SUMO1:Ubc9 complex was 85 ± 30 nM (Fig. 7*F* and Fig. S14*A*), which is similar to 82 ± 23 nM measured previously (27). The binding of M82I-SUMO1: Ubc9 complex was reduced by sevenfold (Kd = 571 ± 134 nM) (Fig. 7*F* and Fig. S14*B*).

### Disruption of Met-aromatic motif leads to inefficient substrate SUMOylation

**Figure 8:**
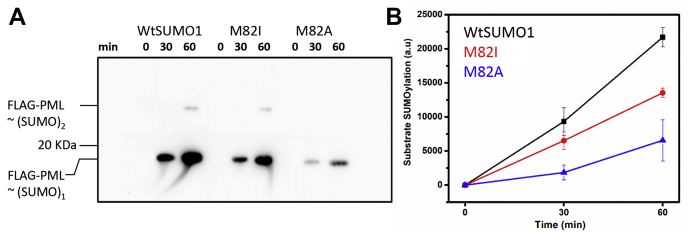
**SUMOylation assay with Wt and mutant SUMO1 proteins.***A*, western blot images of FLAG-PML SUMOylation by Wt and mutant SUMO1 proteins were probed with Anti FLAG antibody. *B*, mono SUMOylation level of FLAG-PML substrate was quantified for Wt, M82I, and M82A SUMO1 mutants and plotted. The FLAG-PML peptide was carefully quantified and used in equal amounts in all the reactions.

SUMOylation assays were carried out to monitor substrate SUMOylation and assess the overall impact upon disruption of methionine-aromatic interaction. PML SUMOylation is essential for forming phase-separated large multiprotein PML nuclear bodies (PML bodies) inside the cell (26). A FLAG-tagged peptide with the consensus SUMOylation motif from PML was used as a substrate for the SUMOylation assays. Individual enzymes (SAE1/SAE2, Ubc9) were purified as described (Materials and methods). SUMOylation was compared between Wt, M82I, and M82A-SUMO1 variants. The reaction was quenched at different time points and run on SDS PAGE. Figure 8*A* shows the western blot image of the SUMOylated fraction of the substrate by individual SUMO variants. The rate of PML SUMOylation was reduced in the M82I-SUMO1 compared with wt-SUMO1 (Fig. 8*B*). A further decrease was seen for M82A SUMO1, highlighting the importance of conserved methionine residue for optimal SUMOylation.

## Discussion

Due to the bulky nature of aromatic amino acids, they form multiple contacts with other amino acids when present at the protein’s buried core. The relevance of geometry in buried aromatic interactions toward the protein’s stability and function is of great interest. Recently, we reported that a conserved edge-to-face interaction between Y51 and F64 at the core of SUMO is critical for its stability and function (28). Interestingly, another aromatic residue (F66) is also conserved in the core, which does not interact with other aromatics in SUMO. Instead, the F66 aromatic ring interacts with a methionine residue (M82). Methionines are hydrophobic due to their aliphatic side chain and are typically buried in a protein’s hydrophobic core. However, methionine is far more interesting than a generic hydrophobic residue because it also includes a sulfur atom in its sidechain. A fascinating property of the sulfur atom is that it can form Met-aromatic noncovalent interactions at a more significant distance (∼5–6 Å) than any other noncovalent interactions in proteins. All-atom MD studies indicate that the energy of such an interaction is up to 3 kJ/mol, equivalent to salt–bridge interactions (13). The relevance of Met-aromatic interactions has been studied in the context of protein–protein complex interfaces, where such interaction was important for binding and function (9). Both methionines and aromatics are prevalent at the protein’s hydrophobic core and buried Met-aromatic interactions are common. We studied whether buried Met-aromatic interactions have any long-range effect on protein structure, stability, and function.

The M82 sulfur atom forms an “up” conformation with the F66 aromatic ring. Multiple sequence alignment revealed significant conservation of the methionine-aromatic pair across all SUMO homologs. We found that the conserved M82–F66 pair is a unique SUMO fold signature that is absent in other Ubls. For other Ubls such as Ubiquitin or NEDD8, a conserved isoleucine is present in a structurally similar position. However, a similar substitution in SUMO with isoleucine perturbs the Met-aromatic interaction and leads to a significant loss of thermodynamic stability. Analysis of the Van't Hoff plot of the thermal melts shows about 1.1 kcal/mol reduction in SUMO stability upon M82I substitution. MD simulation studies also suggest a similar stabilization of ∼0.72 kcal/mol for the Met-Phe pair over the Ile-Phe analog. NMR studies showed that the effect of disruption of Met-aromatic interaction is not localized at the site of perturbation. Instead, the effect is spread across the beta-sheet of SUMO. Although β1 and β2 strands have no direct contact with the methionine M82, CSPs were observed in these strands upon M82I substitution. Intrastrand hydrogen bonds across β1 to β5 connect the beta-sheet in SUMO proteins, and perturbations in one region presumably spread to distant regions through the hydrogen bond network (29).

Given the regulatory nature of SUMOs in several biological processes, their structural integrity is essential for function. Although the buried Met-aromatic motif does not interact with SUMOylation cascade enzymes or SIMs directly, it is critical for SUMO’s structural integrity. All-atom MD simulation suggests that the β3 to β5 loop becomes more open and dynamic when the Met-aromatic interaction is disrupted. The β sheet conformations and cofactor binding interfaces also diverge from the native SUMO fold. Consequently, the affinity of SUMO/SIM and SUMO/Ubc9 complexes decreases. The SIM binding interface and Ubc9 binding interface in SUMO are distinct. The reduced binding for both the cofactors highlights the importance of buried Met-aromatic motif in maintaining native surface topology. Moreover, SUMOylation assay reconfirmed that a stable Met-aromatic interaction is essential for efficient substrate SUMOylation. Our results highlight that conserved Met-aromatic motifs buried at the core are essential for maintaining the protein’s structure–function relationship.

The sulfur atom in methionine can be readily oxidized to form the corresponding sulfoxide, converting an apolar side chain to a polar side chain. The oxidization significantly modulates the Met-aromatic interaction (30, 31). The Met-aromatic interaction in aqueous solution is weakened upon oxidation. The Met-aromatic interaction between M120 from LTα and W107 from TNFR1 in the LTα/TNFR1 complex is inhibited by the oxidation of M120 (30). The current work highlights the relevance of the buried Met-aromatic motif in SUMO and suggests that oxidation of methionines at the buried core may modulate SUMO’s stability and function. SUMO is the central player in cellular SUMO signaling, and its sensitivity to oxidation state implies that SUMO signaling will be a significant sensor of the cellular oxidation state. Indeed SUMO signaling is an exemplary sensor for redox species (32). The SUMOylation pathway is affected by oxidative stress in a variety of ways. Reactive oxygen species (ROS) can inhibit SUMOylation reversibly by inducing a disulfide bond between the catalytic cysteines of the SUMO E1 and SUMO E2 enzymes (33). ROS also oxidizes the deSUMOylation enzyme’s active site cysteine to inactivate them or activate SUMO E3 ligases (34, 35, 36). Our results suggest that oxidative stress may affect the SUMO signaling by modulating the buried Met-aromatic motif in SUMO, which is now an exciting subject of future research.

## Experimental procedures

### Mutagenesis and protein purification

The DNA fragment encoding the 6xHis tagged human wild-type sumo1 in Ampicillin resistant pQE-80L vector was a gift from Dr Sri Rama Koti, TIFR, Mumbai. Substitutions were done using site-directed mutagenesis, and the corresponding clones were verified by sequencing. For overexpression and purification, clones were transformed in BL21 (DE3) bacterial cells and grown in Luria Bertani (LB) broth using the standard procedure. For NMR experiments, uniformly ^13^C/^15^N-labeled samples were prepared by growing the bacteria in an M9 medium containing ^15^NH_4_Cl and ^13^C_6_-glucose. For the preparation of uniformly ^15^N-labeled samples, ^13^C_6_-glucose was replaced by unlabeled d-glucose. Cells were grown at 37 °C, and protein expression was induced at OD_600_ of 0.8 by adding IPTG (isopropyl this-β-d-thiogalactoside) final concentration of 1 mM. After another 5 h of growth, the cells were harvested by centrifugation, resuspended in the lysis buffer (50 mM Na_2_HPO_4_, 25 mM imidazole (pH 8.0), 300 mM NaCl), and lysed by sonication. The lysate’s centrifuged supernatant was mixed with pre-equilibrated Ni^2+^ NTA-agarose beads (Protino) for 2 h. The slurry mixture (lysate with beads) was loaded to an open column, washed, and eluted with different imidazole concentrations present in the lysis buffer (pH 8.0). Further purification was done by gel filtration (Superdex 75 16/600) column. The final protein was obtained in PBS containing 1 mM DTT at pH 7.4. For NMR experiments, the protein sample was supplemented by 10% D_2_O.

### MD simulations

The starting structure for all-atom Molecular Dynamics (MD) simulations of Wt human SUMO1was obtained from 1.35 Å resolution crystal structure (4WJO) (21). Methionine 82 mutant SUMO1 structures were generated by replacing the specific side chain with the best aligning rotamer from Dunbrack rotamer library (37) inbuilt in the molecular visualization program UCSF Chimera (38). Water molecules present in the crystal structure were removed. Unbiased MD simulations were performed in GROMACS version 5.1.2 (www.Gromacs.org) (39, 40). All simulations were carried out using AMBER99SB-ILDN force field (41, 42). All acidic and basic residues were modeled in their charge states. The initial structures were solvated in explicit TIP3P water model (43) in an appropriate box. Each system was neutralized by adding counter ions. Unfavorable interactions in each system were relaxed by energy minimization using the steepest descent minimization algorithm for 5000 steps until the maximum force on each atom was less than 1000 kJ/mol, followed by sequential equilibration in NVT and NPT ensemble. The solvent density was adjusted under periodic boundary conditions under isobaric and isothermal conditions at a pressure of 1 bar and a temperature of 300K. Temperature control was achieved using a V-rescale modified Berendsen thermostat (44) with a time constant of 0.1 ps during both NVT and NPT equilibration and a time constant of 2.5 ps production steps. Pressure control was achieved using Parrinello–Rahman barostat (45) with a time constant of 0.1 ps during NPT equilibration and 5 ps during production steps, respectively. The system was equilibrated in an NPT ensemble for 600 ps with a 2 fs simulation time step. Final production runs were carried out on the NPT ensemble for 100 ns with a 3 fs time step. The long-range electrostatic interaction was calculated using the smooth particle Mesh Ewald sum method (46, 47) with a cutoff of 1 nm. van der Waals and short-range interactions were terminated at 1 nm cutoff. Every bond length was constrained using LINCS algorithm (48, 49).

### Circular dichroism measurements

Circular dichroism (CD) measurements were carried out on a Jasco J-1500 spectrometer. Far UV protein scans and thermal melt experiments were recorded for the 15 μM concentration of protein in PBS at pH 7.4 in a 1 mm path length cuvette. An average of five scans at 20 °C with 50 nm per minute scan speed and 1 s of digital integration time were plotted for the graph. For thermal melts, mean residue ellipticity at 222 nm wavelength was monitored from 20 to 90 °C with a rate of 1 degree per minute increase of temperature and 32 s of data integration time and fitted with a two-state folding curve to obtain the Tm of individual mutants and wt-SUMO1. Analysis of the Van't Hoff plot of the thermal melts yielded unfolding free energy of 3.72 kcal/mol for wt-SUMO1 and 2.62 kcal/mol for M82I SUMO1.

### ANS fluorescence measurements

In total, 20 μM of protein and 200 μM of ANS in PBS were used for ANS fluorescence measurement for each protein sample, and data were recorded on Fluoromax-4 (Horiba Jobin Yvon) spectrofluorometer. Samples were excited at 380 nm (Slit width 5 nm), and emission spectra were recorded from 385 nm to 650 nm (Slit width 10 nm) at 25 °C. Averages of three independent scans are reported in Figure 2*C*.

### Synthetic peptides

All the synthetic peptides were purchased from Lifetein LLC as lyophilized powders. The peptides were subsequently dissolved in PBS and used for titration by NMR.

### NMR experiments

NMR spectra were recorded at 298K on 800 MHz Bruker Avance III HD spectrometer with a cryo-probehead, processed with NMRpipe (50)^,^ and analyzed in NMRFAM-SPARKY (51). Standard triple resonance CBCA(CO)NH, HNCACB, HNCO, and HN(CA)CO experiments were used for backbone assignments. All NMR data for resonance assignment were processed using NMRPipe and analyzed using Sparky software (52). Following peak picking of the backbone experimental data in Sparky, the data were assigned by the PINE NMR-server (53) and then verified, corrected, and completed manually. All NMR titrations were carried out at 298K in PBS buffer, pH 7.4. NMRFAM-SPARKY was used to calculate the peak intensities. The graphs were plotted using the Origin software package.

### ITC experiments

ITC experiments were performed in the GE ITC200 instrument. Wt SUMO1, M82I SUMO1, and Ubc9 proteins were dialyzed overnight at 4 °C in buffer containing 25 mM Tris, pH 8.0, and 2 mM BME. Wt and mutant SUMOs were kept in cells and Ubc9 in the syringe. The concentrations of wt-SUMO1 and M82I-SUMO1 proteins were 12 μM and 7 μM, respectively. The concentration of Ubc9 was 365 μM. The temperature was set to 298 K.

### SUMOylation assay

For SUMOylation assays, 5 μM substrate (FLAG-tagged PML peptide with SUMOylation motif) and 5 μM Wt or mutant SUMO1 were incubated with 1 μM E1 and 2.5 μM E2. The reaction was started by adding 1 mM ATP. Reaction was carried out at room temperature in buffer containing 25 mM Tris pH 8.5, 150 mM NaCl, 5 mM MgCl_2_, 0.1% Tween 20. The reaction was run on 12% SDS PAGE and blotted against the Anti-FLAG antibody. Images were quantified on ImageJ (54).

## Data availibility

The backbone assignment of M82I SUMO1 has been submitted to BMRB with accession ID 50949. All the other data are contained within the article.

## Supporting information

This article contains supporting information.

## Conflicts of interest

The authors declare that they have no conflicts of interest with the contents of this article.
