# Supplementary material for: An “up” oriented methionine-aromatic structural motif in SUMO is critical for its stability and activity
Source: J Biol Chem. 2021 Jul 15;297(2):100970. doi: 10.1016/j.jbc.2021.100970 (PMC8353491; doi:10.1016/j.jbc.2021.100970)
Supplement: Supplemental Figures S1–S14 [file mmc1.pdf]

Supporting Information for

An 'up' oriented methionine-aromatic structural motif in SUMO is critical for its stability and activity

Kiran Sankar Chatterjee[1], and Ranabir Das[1,2]

From the [1]National Centre for Biological Sciences, Tata Institute of Fundamental Research, Bengaluru-560065, India

[2]To whom correspondence should be addressed: Ranabir Das, National Centre for Biological Sciences, Tata Institute of Fundamental Research, Bengaluru-560065, India. rana@ncbs.res.in ; Phone: +91-80-23666105/+91-80-23666545/+91-20-25908008; Fax +91-80-23636662

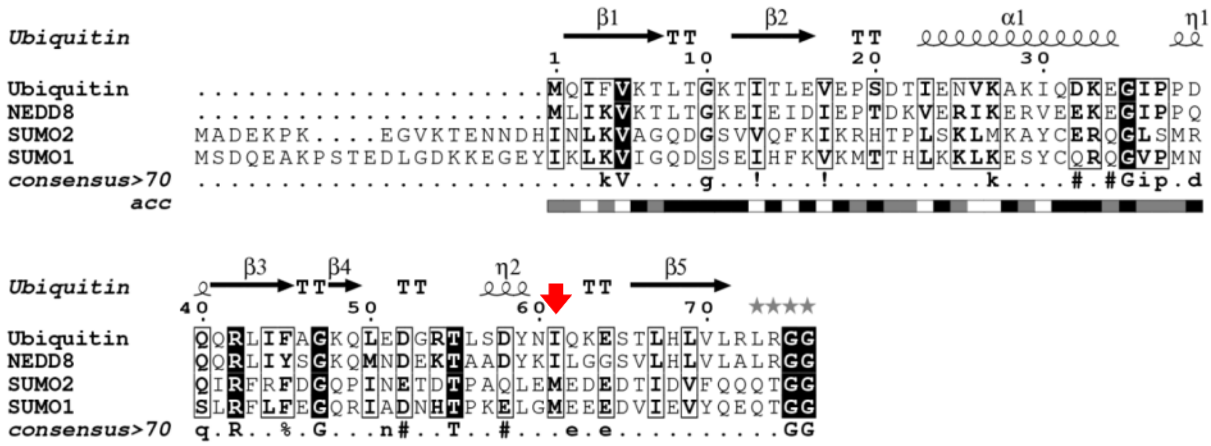

Figure S1: Sequence Alignment of human Ubiquitin-like modifiers (Ubls). Conserved Methionine position in SUMOs is highlighted with a red arrow. Solvent accessibility is shown at the bottom of alignment in a color scale from black to white, where black indicates solvent-exposed and white represents buried residues. Corresponding secondary structure against the primary sequence is aligned on top.

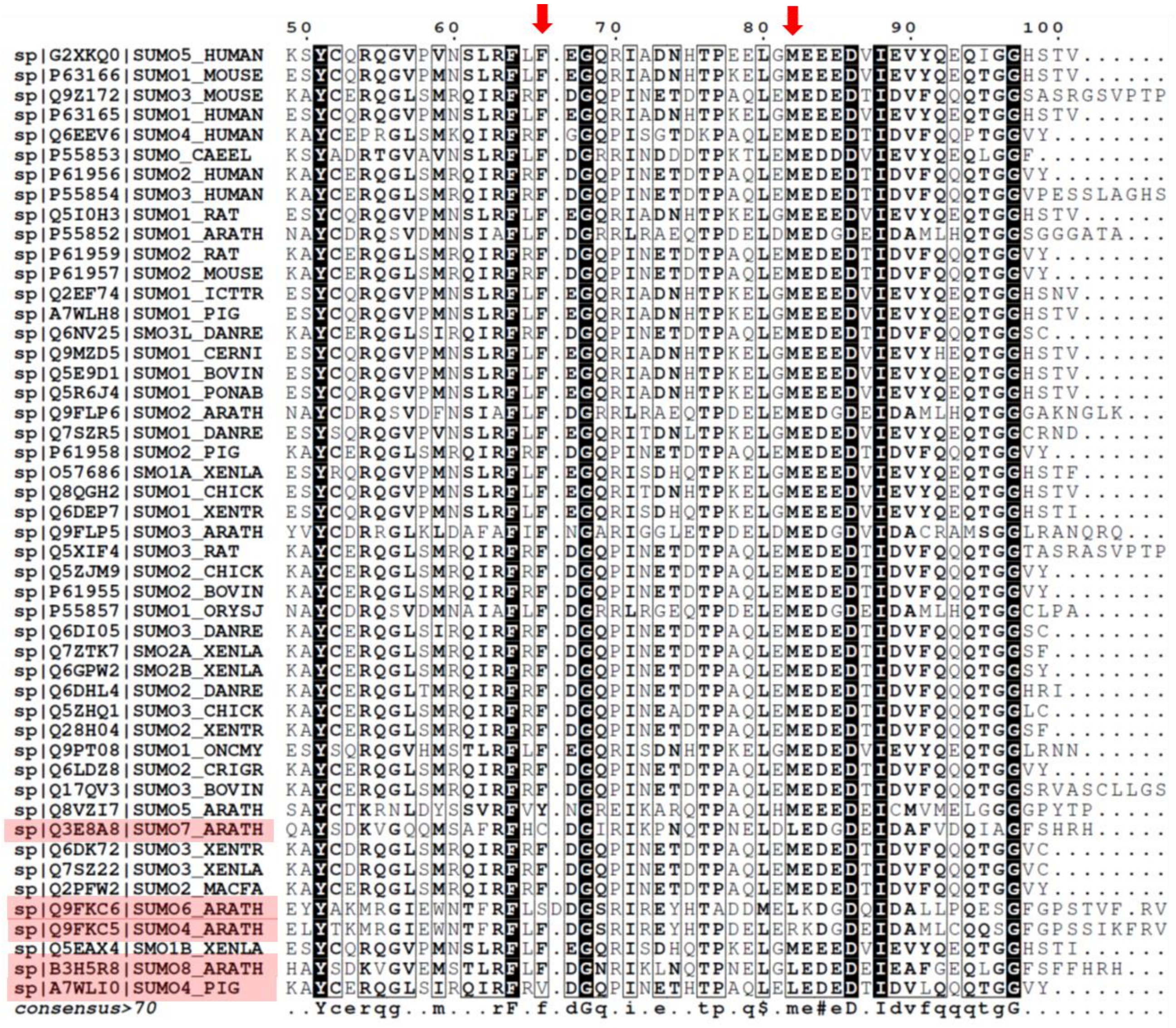

Figure S2: Multiple Sequence Alignment of all curated SUMO sequences from uniport database. Conserved methionine and phenylalanine position in SUMOs is highlighted with a red arrow on top. Solvent accessibility is shown in background in a color scale from black to white, where black indicates solvent-exposed, and white represents buried residues. The alignment is made against human SUMO1 sequence. Five SUMO sequences where methionine-aromatic interaction is not conserved are highlighted in red box. Out of five such sequences SUMO6, SUMO7 and SUMO8 from Arabidopsis are putative SUMO sequences and need further validations.

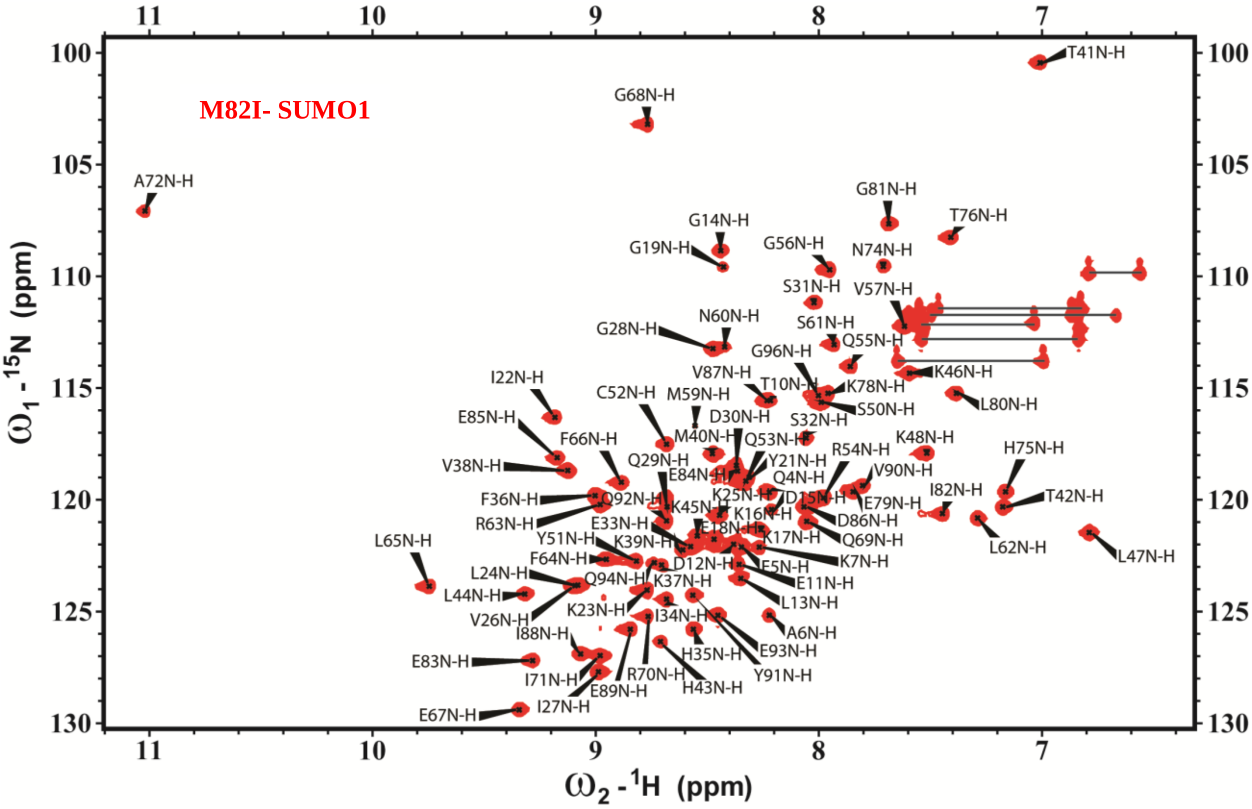

Figure S3: ^{15}N-^{1}H edited HSQC Spectrum of uniformly labelled ^{15}N protein sample of M82I SUMO1 in PBS, pH 7.4. The backbone amide assignments are labelled beside the peaks. The Glutamine and Asparagine sidechains are connected by grey horizontal lines.

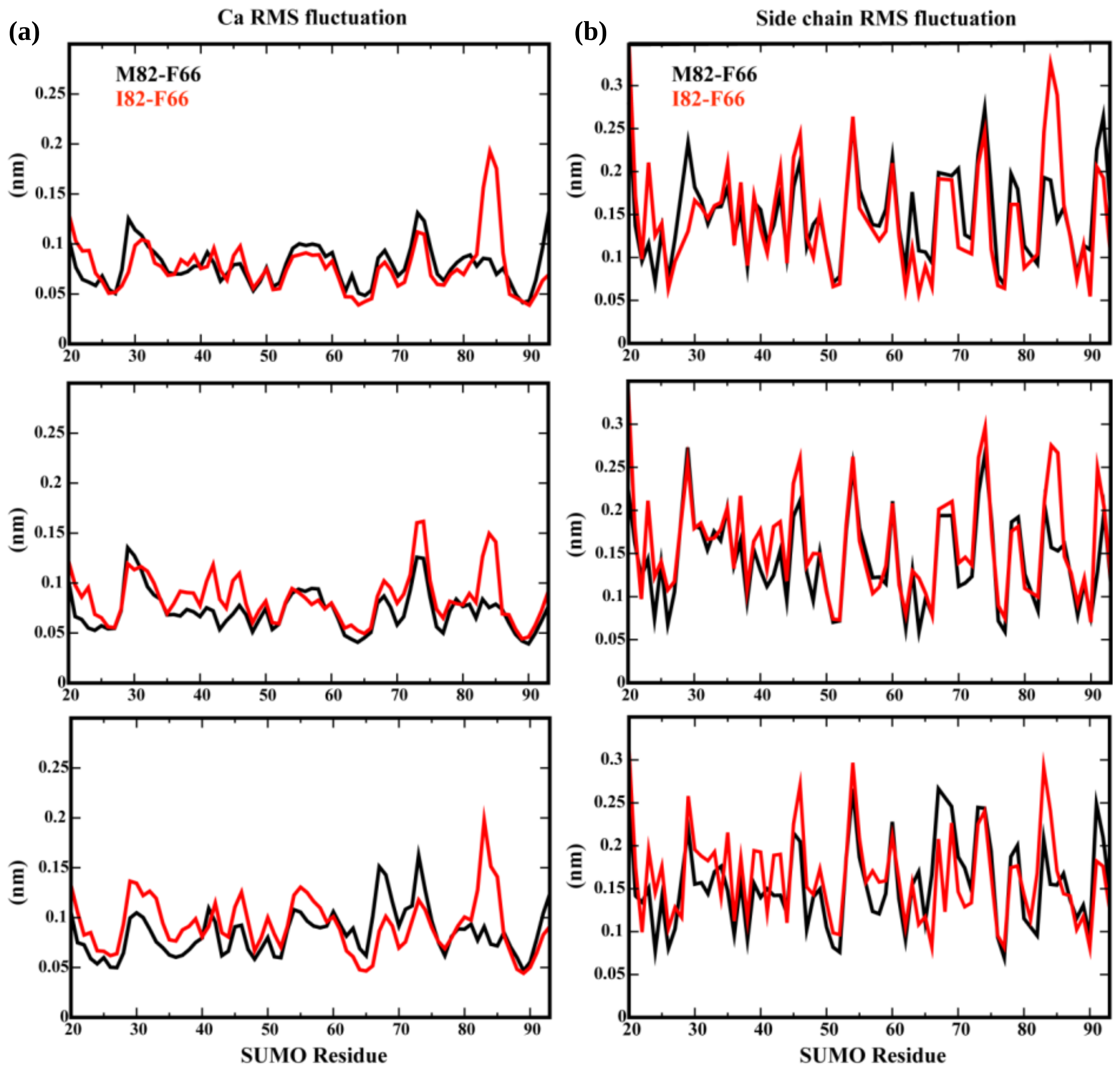

Figure S4: Effect on backbone and side chain dynamics upon Methionine to Isoleucine substitution in SUMO. (a) Backbone Cα RMS fluctuation of Wt and M82I SUMO1 for three independent runs. Wt is shown in Black and M82I in Red. (b) Side chain RMS fluctuation of Wt and M82I SUMO1 for three independent runs. Wt is shown in Black and M82I in Red.

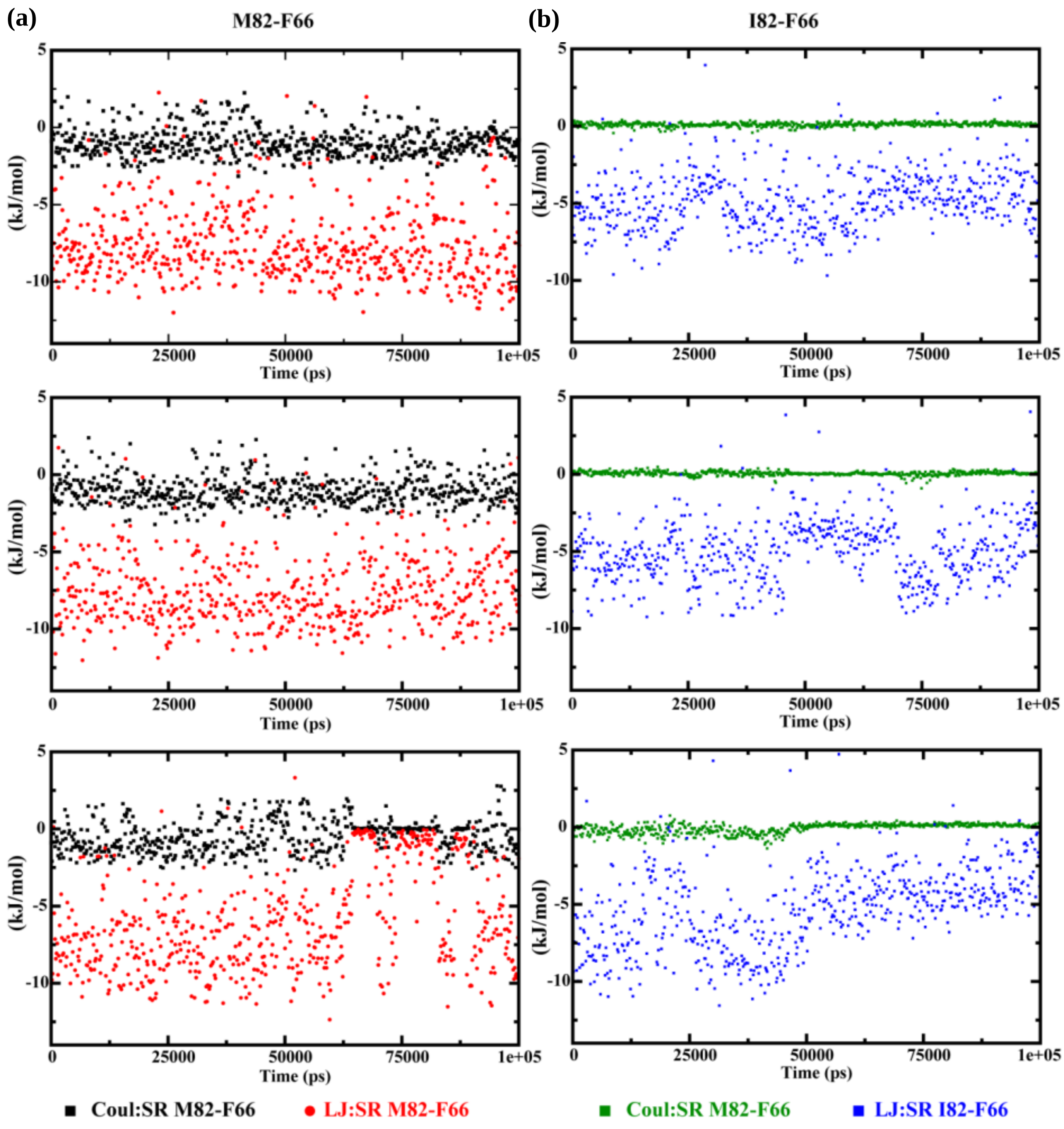

Figure S5: Comparison of interaction energies between M82-F66 and I82-F66 pairs. (a) Electrostatic (Coul:SR) and van der Waals (LJ:SR) interaction energy of M82-F66 pair calculated for three independent trajectories. (b) Electrostatic and van der Waals interaction energies of I82-F66 pair plotted for three independent trajectories.

	M82-F66 (Wt) (kJ/mol)		I82-F66 (M82I) (kJ/mol)
**Run1**	**Coul-SR**	**-1.02**	**0.11**
	**LJ-SR**	**-7.61**	**-4.86**
**Run2**	**Coul-SR**	**-0.99**	**0.048**
	**LJ-SR**	**-7.59**	**-4.96**
**Run3**	**Coul-SR**	**-0.53**	**-0.05**
	**LJ-SR**	**-5.87**	**-5.21**

Table S6: Interaction energies of M82-F66 and I82-F66 pair calculated over three independent simulations of 100 ns each. Backbone atoms of the interacting pair were not included in the calculation.

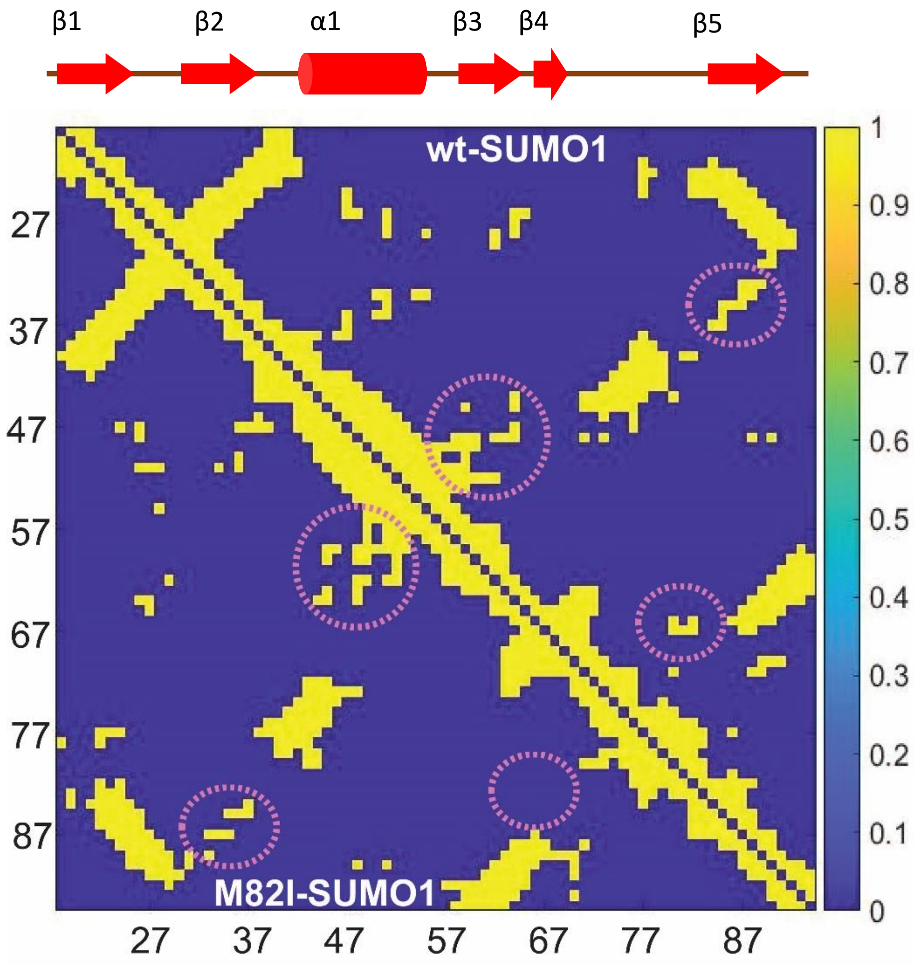

Figure S7: A comparison of M82I-SUMO1 and wt-SUMO1 structures taken at the same time frame of individual all atom MD simulation trajectories. The contact map of wt-SUMO1 and M82I-SUMO1 is displayed in the same 2D plot, where wt-SUMO1 is represented in the upper right, and the M82I is represented in the lower left region of the plot. The differences between the contacts are circled.

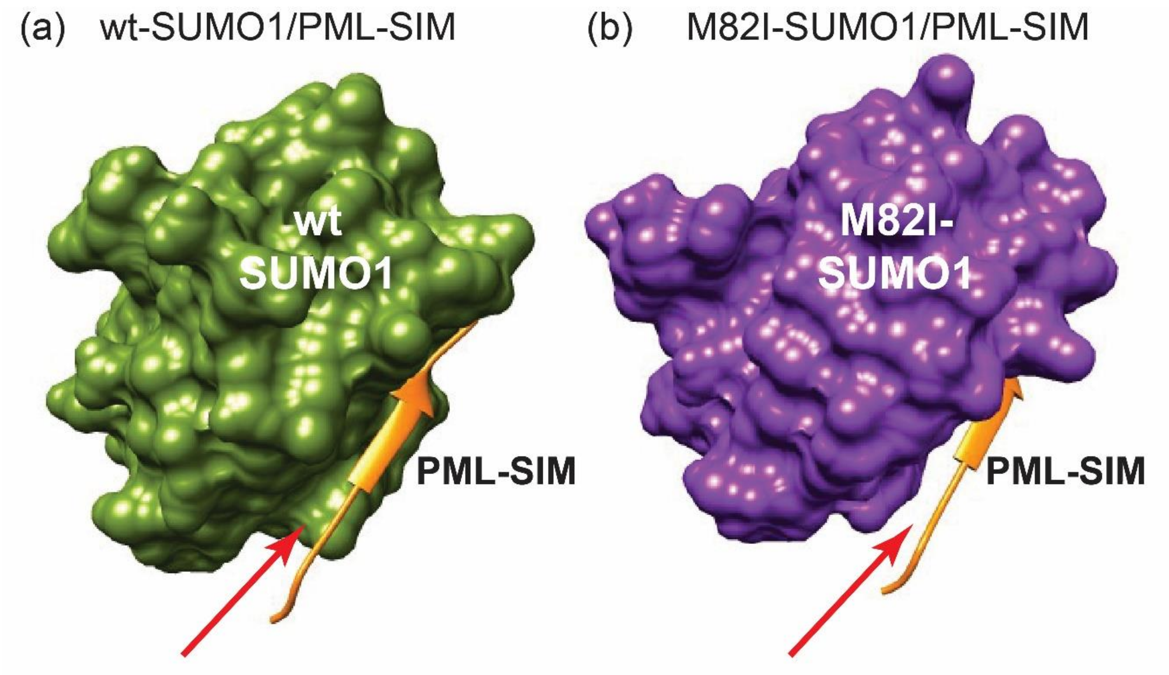

Figure S8: The models of (a) wt-SUMO1/PML-SIM and (b) M82I-SUMO1/PML-SIM complex. The SUMO1/PML-SIM complex (PDB id: 2WJO) was used for modeling. The region where M82I substitution causes loss of contacts between SUMO1 and PML-SIM is shown by red arrows.

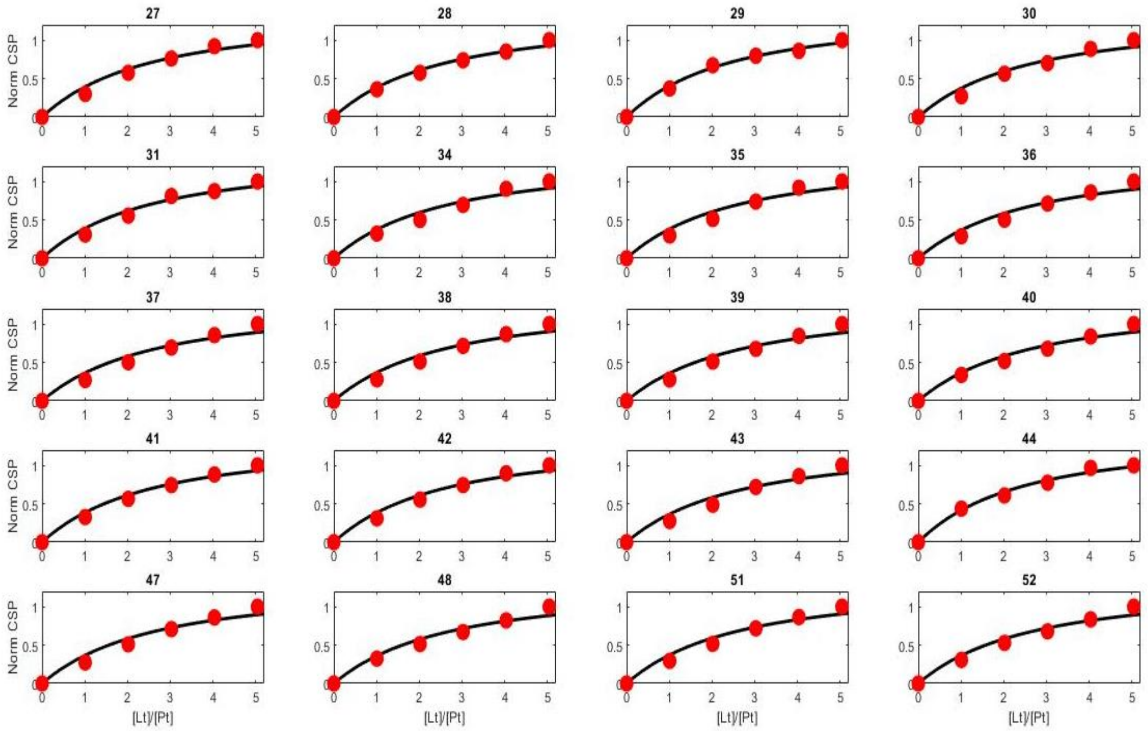

Figure S9: The fit of M82I-SUMO1 peak shifts against the concentration ratio [PML-SIM]/[M82I-SUMO1] yielded the K_d of the M82I-SUMO1/PML-SIM complex (568±40 μM). The fit of twenty residues are shown, and the residue numbers are given on the top of every fit.

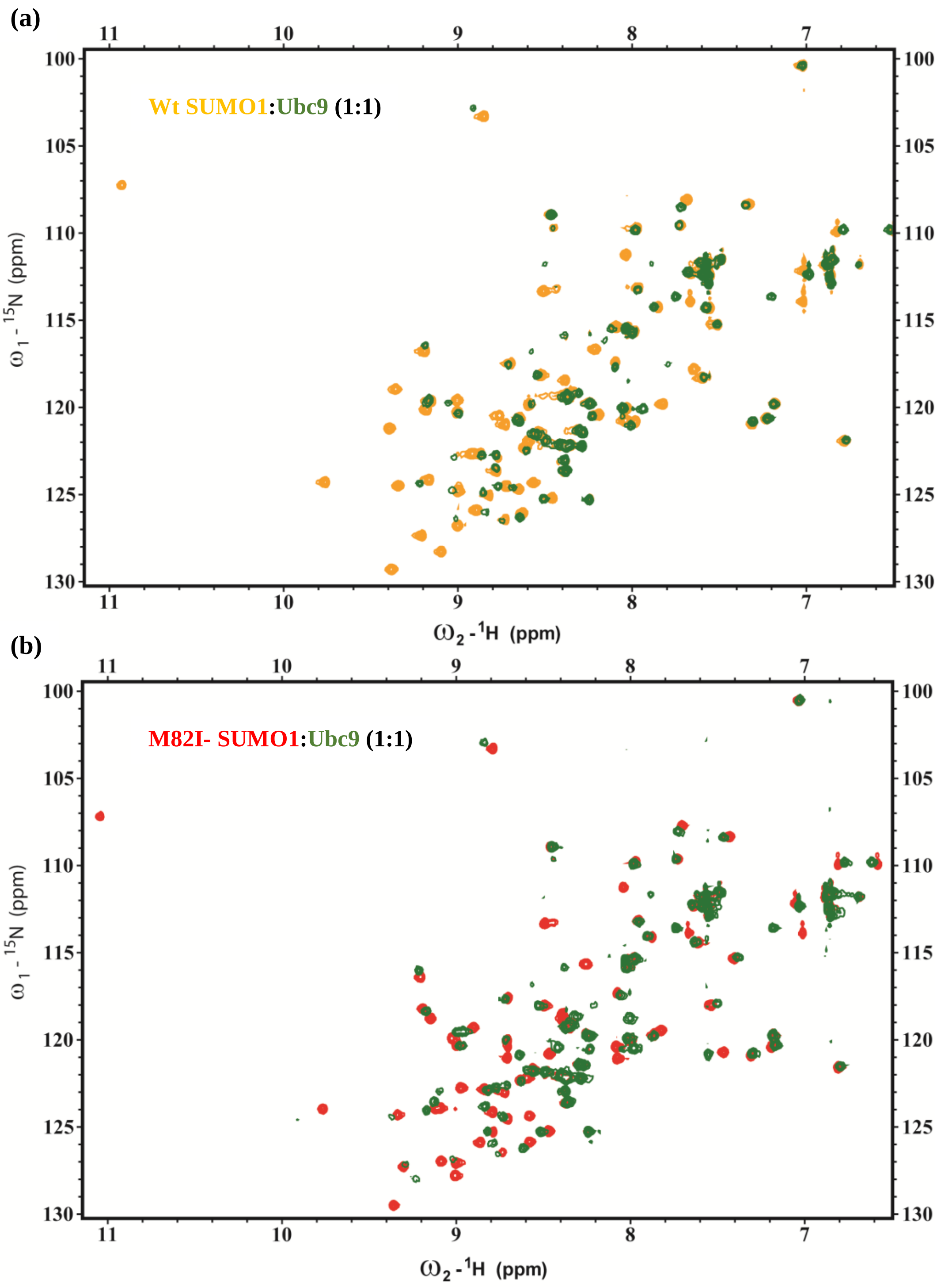

Figure S10: Interaction of Wt and M82I-SUMO1 with Ubc9. (a) Overlays of ^{15}N-^{1}H edited HSQC spectra of wt-SUMO1 apo (Yellow) and Ubc9 bound (1:1) (Green) are shown. (b) Overlays of ^{15}N-^{1}H edited HSQC spectra of M82I-SUMO1 in apo (Red) and holo (Ubc9 bound) form (1:1) (Green).

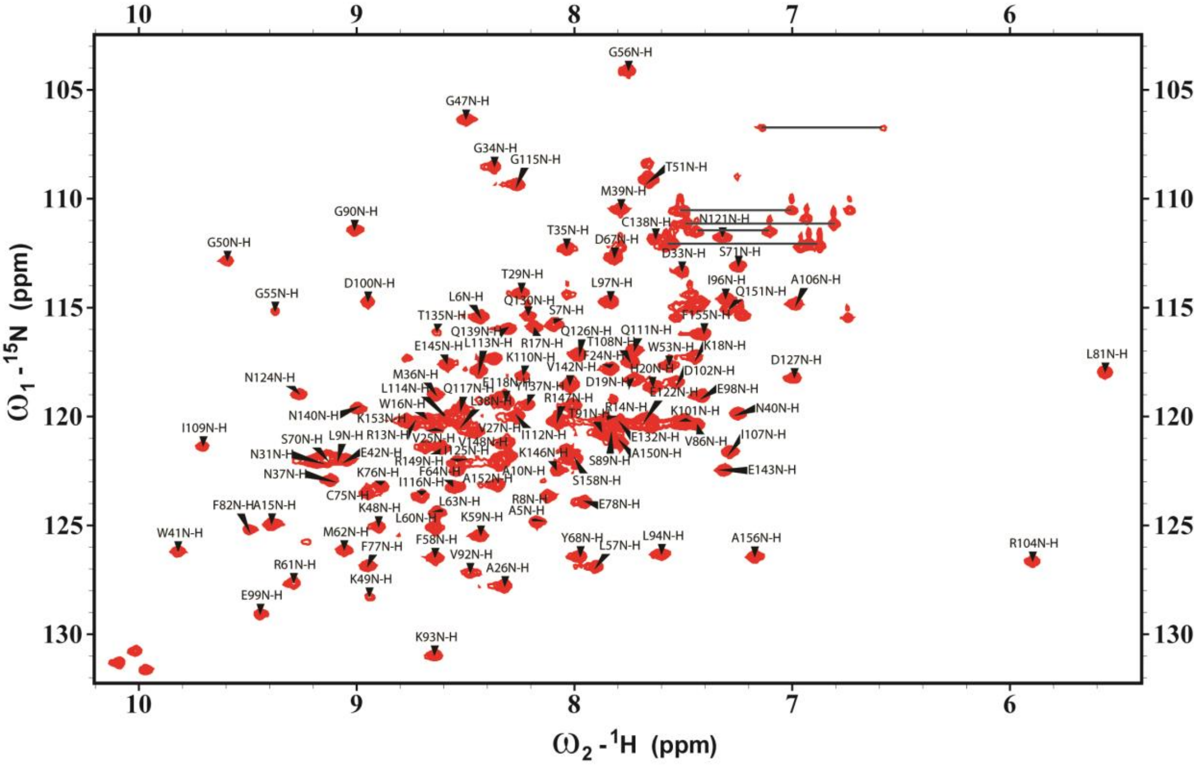

Figure S11: ^{15}N-^{1}H edited HSQC Spectrum of uniformly labelled ^{15}N protein sample of Ubc9 (C93K) in PBS, pH 7.4. The backbone amide assignments are labelled beside the peaks. The Glutamine and Asparagine sidechains are connected by grey horizontal lines.

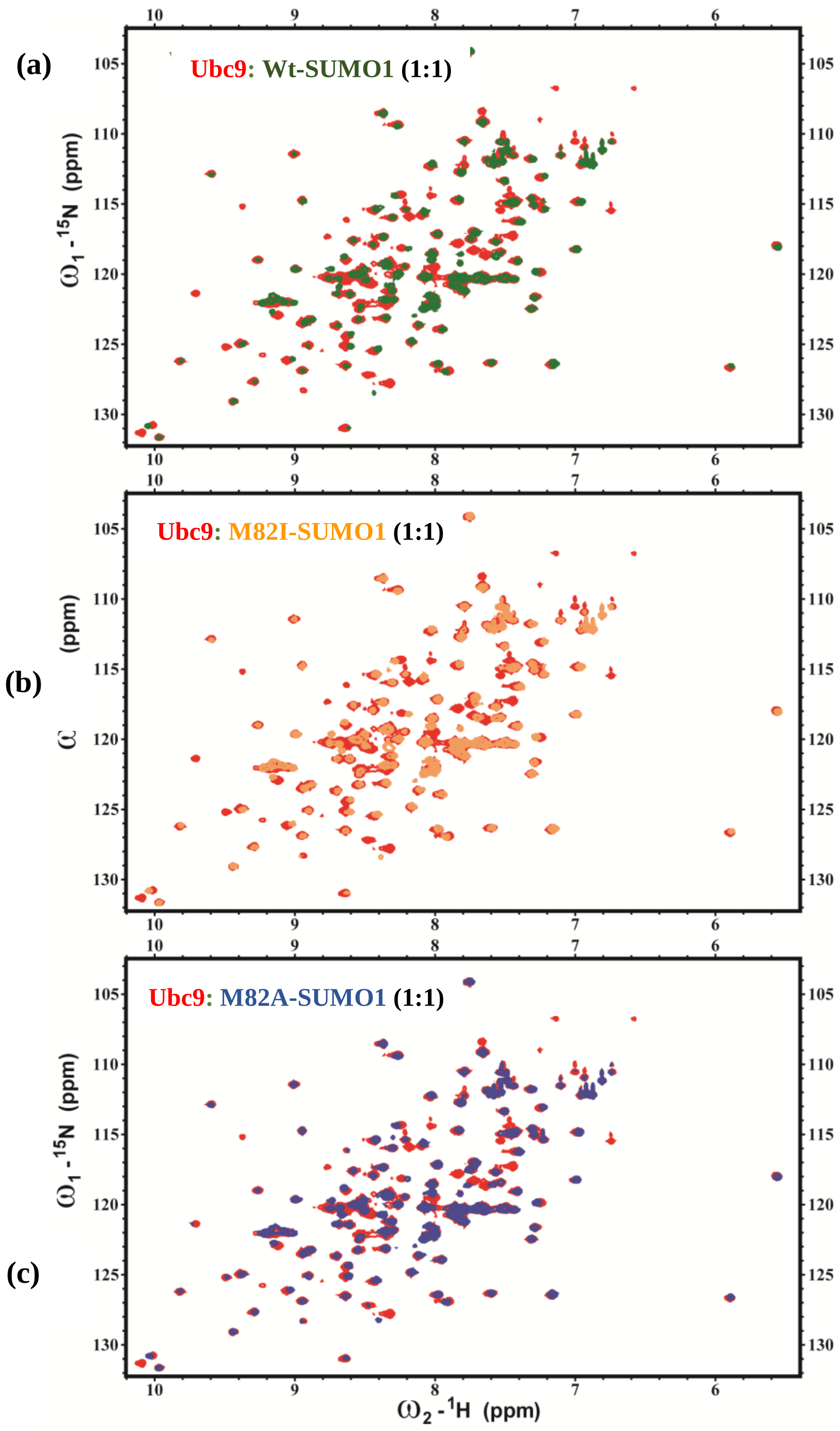

Figure S12: Interaction of Ubc9 with SUMO1 variants. Overlays of ^{15}N-^{1}H edited HSQC Spectrum of Ubc9 bound SUMO variants (1:1) on apo Ubc9 (C93K) (Red) (in PBS) are shown. (a) Wt SUMO1 in Green, (b) M82I in Orange and (c) M82A SUMO1 in Blue. Upon interaction with SUMO1 multiple peaks of Ubc9 broadens out. Effect of broadening is most for Wt SUMO1/Ubc9 interaction and least for M82A SUMO1/Ubc9 pair.

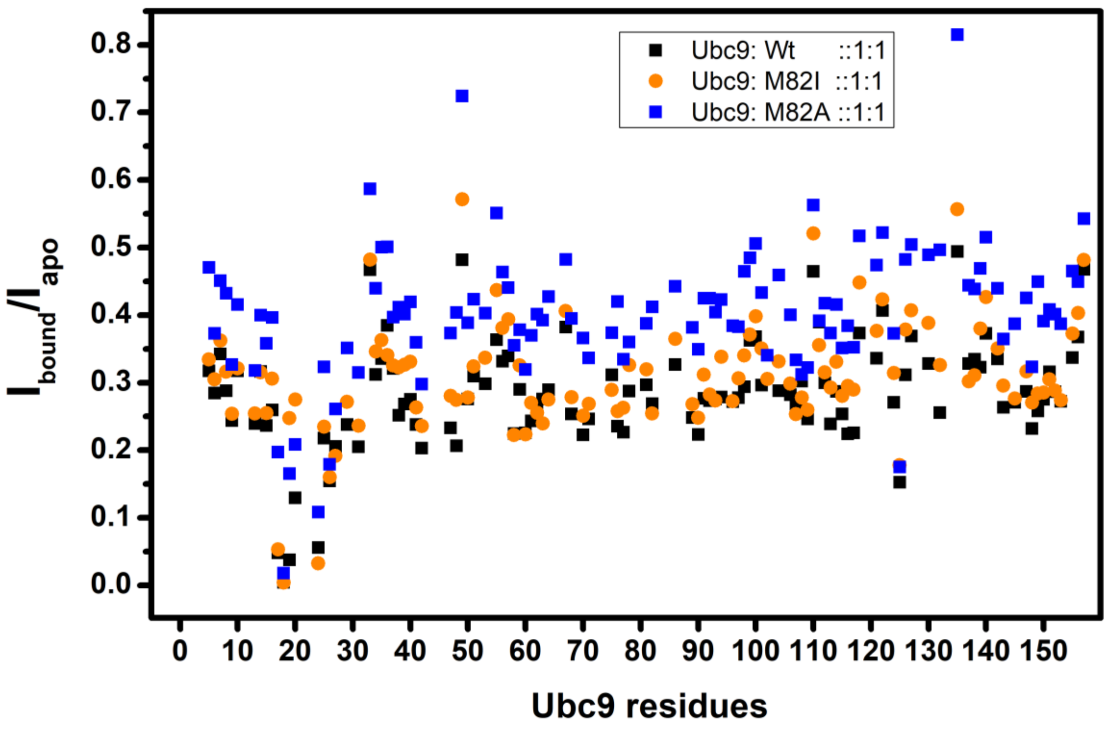

Figure S13: Comparison of Intensity ratio **I_{bound}/I_{apo}** of Ubc9 backbone amide resonances upon interactions with Methionine variant SUMO1s at 1:1 stoichiometry. A progressively higher **I_{bound}/I_{apo}** ratio from Wt SUMO1 < M82I < M82A suggests lesser binding of SUMO upon disruption of core Met-aromatic interaction.

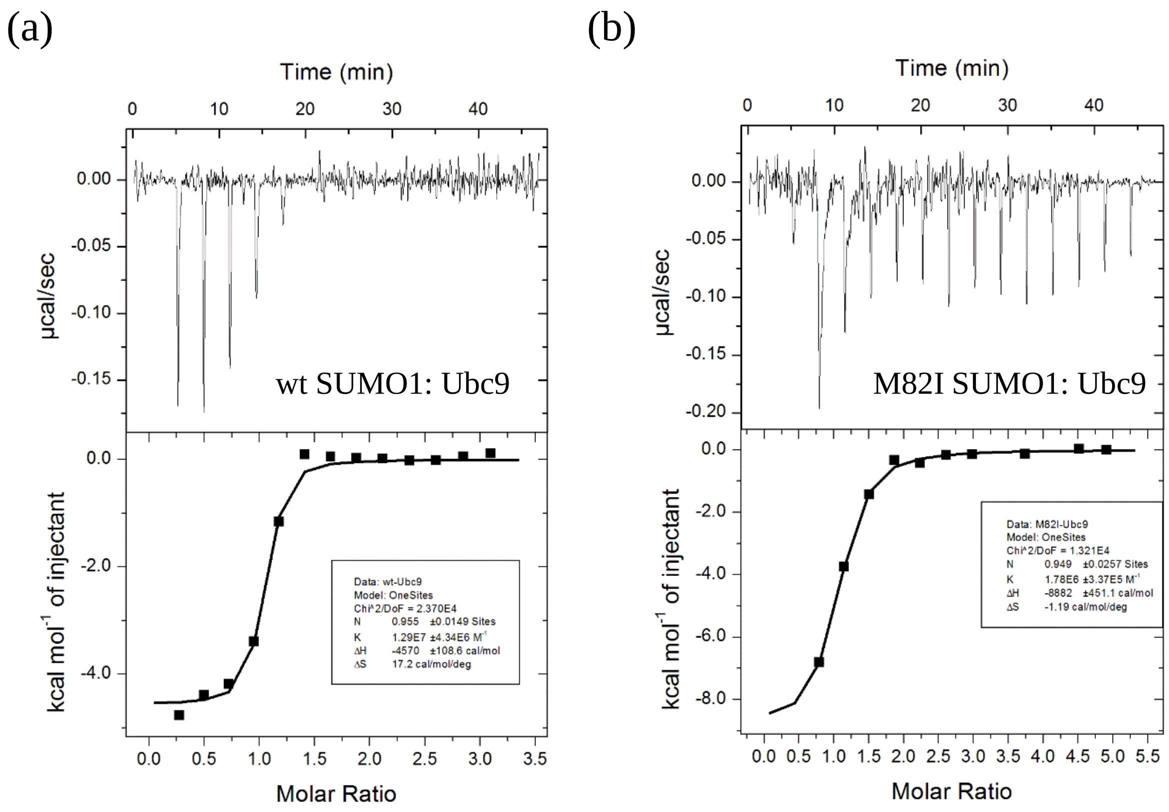

Figure S14: Interaction of wt- and M82I-SUMO1 with Ubc9 measured using Isothermal Titration calorimetry. (a) wt-SUMO1 and (b) M82I-SUMO1.
